# Sequence based prediction of enhancer regions from DNA random walk

**DOI:** 10.1038/s41598-018-33413-y

**Published:** 2018-10-29

**Authors:** Anand Pratap Singh, Sarthak Mishra, Suraiya Jabin

**Affiliations:** Department of Computer Science, Jamia Millia Islamia, Jamia Nagar, 110025 New Delhi India

## Abstract

Regulatory elements play a critical role in development process of eukaryotic organisms by controlling the spatio-temporal pattern of gene expression. Enhancer is one of these elements which contributes to the regulation of gene expression through chromatin loop or eRNA expression. Experimental identification of a novel enhancer is a costly exercise, due to which there is an interest in computational approaches to predict enhancer regions in a genome. Existing computational approaches to achieve this goal have primarily been based on training of high-throughput data such as transcription factor binding sites (TFBS), DNA methylation, and histone modification marks etc. On the other hand, purely sequence based approaches to predict enhancer regions are promising as they are not biased by the complexity or context specificity of such datasets. In sequence based approaches, machine learning models are either directly trained on sequences or sequence features, to classify sequences as enhancers or non-enhancers. In this paper, we derived statistical and nonlinear dynamic features along with k-mer features from experimentally validated sequences taken from Vista Enhancer Browser through random walk model and applied different machine learning based methods to predict whether an input test sequence is enhancer or not. Experimental results demonstrate the success of proposed model based on Ensemble method with area under curve (AUC) 0.86, 0.89, and 0.87 in B cells, T cells, and Natural killer cells for histone marks dataset.

## Introduction

More than 98% of the human genome constitutes the non-coding region with most of the regulatory elements falling in this region. Regulatory region plays an important role in gene regulation and it occupies a centre stage in understanding the gene expression^1^. Regulatory regions do not code for proteins instead control the expression of other coding regions. These regions can be classified as promoter, enhancer, silencer, and insulator etc. Promoters occur in the vicinity of coding regions and they bind to transcription factor protein that initiates DNA transcription^2^. Enhancers are regions situated distant from transcription start site. These can not only be found upstream or downstream of gene but also within introns^3^. The identification of novel enhancers is a challenging task for several reasons. First, number of enhancer sequences is very small as compared to the size of human genome. Second, their location relative to their target gene (or genes) is highly variable as they do not necessarily act on the respective closest promoter but can bypass neighbouring genes, to regulate genes, located more distantly along a chromosome. Third, in contrast to the well-defined sequence code of protein-coding genes, the general sequence code of enhancers, if one exists at all, is poorly understood^4^. Enhancers share core properties with promoters but the RNA produced by them may differ. Enhancers produce eRNA, which are sensitive to exome-mediated decay. They are relatively short, unspliced, non-polyadenylated, and are retained in the nucleus. Whereas, promoter upstream antisense transcripts (PROMPTs) are heterologous in length and they are produced only upstream of the promoters of active protein coding genes. Enhancers and promoters are similar in having transcription factor binding sites. Enhancers play an important role during development and in the regulation of cellular processes during an organism’s lifetime^2^. They behave in cell specific manner; few enhancers are active in differentiated cell at a particular time, while the others are in an inactive state^3^. This feature of enhancer makes it a good candidate to differentiate cell types.

*De novo* characterization of an enhancer is a challenging task, despite constantly reducing cost of performing site directed mutagenesis and analysis of its transcriptional impact. As non-coding DNA are present in high proportion in eukaryotes, computational methods to identify novel enhancers have become handy to filter candidates from the non-coding regions. This problem of enhancer prediction can be simply stated as: Given a DNA sequence, determine if it can function as enhancer^5^. Various computational methods have been used with different features or combination of features to characterize enhancer regions. These features primarily categorize DNA sequences with three sets of properties namely genomic sequence conservation, histone marks, TFBS, and high-resolution analysis^6,7^. Both traditional and Deep learning^8^ based algorithms have been used for predicting enhancers from genomic features or sequences alone. For example, an integrated approach by combining multiple datasets was developed by deriving feature vectors and then making use of these feature vectors to train machine learning based models to predict enhancers^8–18^. Similarly, histone marks like H3K4me3, H3K4me1, and H3K27ac have been used as positive set to classify through ensemble techniques like Adaboost and random forest^13,16^. These features are not flawless and have some serious limitations. For example, it is now known that the enhancer regions are not particularly conserved. Similarly, the presence of histone marks and occurrence of TFBS do not assure the site is an enhancer region^19,20^. Problem is further complicated as all enhancer regions are not available for binding in different cell types^21^, leading to inconsistencies with genomic features described in these methods. Enhancer identification using computational methods is a tricky task, as different kinds of challenges are associated with it. For example, enhancers are not evolutionarily conserved, so evolutionary methods do not help much. Enhancer sequences vary in length and location is not fixed (spread over intergenic region, also it can be found in intronic regions). Transcription factor binding sites also face conservation problem and transcription factors are not specific to enhancers. The framework, used by existing computational methods, includes integration of NGS data, creation of feature vector, and prediction of annotation (enhancer or non-enhancer). The feature vector contains transformed values from TFBS, CHiP-Seq (Chromatin immunoprecipitation with massively parallel DNA sequencing) data^22^, chromatin accessibility information derived from DNase-I hypersensitivity sites or any combination of these datasets. Methods which use mostly NGS data (Chip-seq, DHS) or TFBS data show good prediction but are limited to specific cell types. These methods have lower generalization capability if given unknown cell data e.g., CSI-ANN (based on artificial neural network)^23^, RFECS (random forest)^16^, and DELTA (Adaboost)^13^. Some methods used combination of sequence features, TFBS and NGS data to attain generalization and better accuracy e.g., EMERGE (elastic logistic regression)^24^, PEDLA (deep Neural network)^10^ and EnhancerDB (Deep Belief network)^25^.

To overcome some of the inconsistencies in genomic feature based prediction of enhancers, sequence features can be readily used, as they offer the advantage of overcoming the limitation of cell type specific enhancers^8,12^. Histone marks data is cell or tissue specific and if we train a machine learning based model on histone dataset, naturally the model tends to become biased towards that specific cell type for which it is trained and it may fail badly to do correct predictions for other cell types. By preparing training dataset using ‘gold standard enhancers’ from VISTA Enhancer Browser^26^ for the proposed model, we attempted to solve problem of classifying DNA sequences into enhancers and non-enhancers irrespective of tissue specificity, and the results suggest the success of the proposed model. The common features used in sequence based approaches are k-mer^17^ frequency but as the length of k-mer increases, the frequency of k-mer decreases progressively, while the dimensionality of the model grows [8, 11, and 13]. As soon as the length of k-mer string crosses 6, the frequency matrix may become really sparse which results in overfitting of the machine learning based model^14^. For the proposed work, we calculated feature vector based on k-mer with length ranging from 1 to 6 along with statistical and nonlinear features calculated from DNA random walk model. In this paper, we investigate if sequence features obtained on the basis of DNA random walk model and k-mer features can be used as an alternative model for predicting enhancer regions and it looked promising as long range correlations have also been observed in non-coding DNA sequences in previous studies^15^. DNA random walk models show promises to classify regulatory sequences. The proposed method captured dynamics of DNA through one dimensional random walk by using purine-pyrimidine model. We developed machine learning model based on sequence features extracted from DNA random walk model at genomic scale along with k-mer features. Bagged Tree based Ensemble method outperformed other machine learning methods such as Quadratic Support Vector Machine (SVM)^23,27^, Levenberg-Marquardt Backpropagation^28^ and Bayesian regularized Feed-forward Neural Network (FFNN)^29^ in classification task of enhancers. The other methods that performed comparable are Convolutional Neural Network (CNN)^30–32^, and RUSBoosted Tree based Ensemble method^33^ which is a hybrid sampling/boosting algorithm. The proposed method overcomes the limitation of other enhancer prediction methods, as combination of sequence features improves prediction accuracy. This method is not at all dependent on histone marks, so we hypothesize to predict more complete set of enhancers. The proposed method is an attempt to classify enhancer sequences with better accuracy and generalizability than the existing methods in literature.

## Materials and Methods

**Figure 1:**
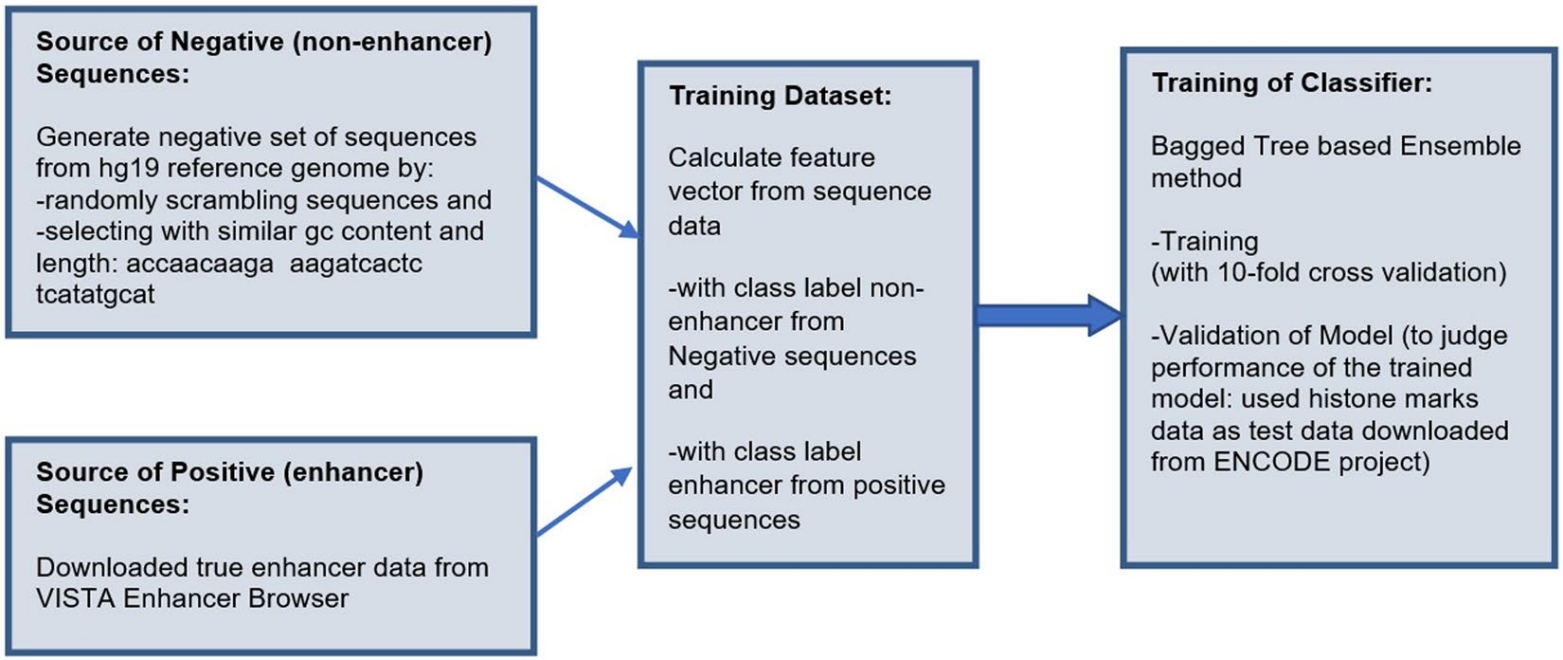
Flow Chart of the Proposed system.

The Fig. 1 shows the flow chart of the proposed system and the following sub-sections explain the system.

### Sources of Datasets

In total, 1798 experimentally validated human non-coding elements with gene enhancer activity, as assessed in human cell lines were collected from the VISTA Enhancer Browser^26^. From non-coding fragments obtained, 900 elements were defined as enhancers that exhibit reproducible pattern in at least three different embryos, whereas 898 elements were defined as enhancers that exhibit non-reproducible pattern in at least three different embryos. These 898 elements are considered as weak enhancers.

The positive set i.e., the set of enhancer sequences, is constructed using the experimentally validated human non-coding fragments taken from the VISTA Enhancer Browser. The length of validated human enhancer ranged from 428 to 8061 base pairs (bp) with median of 1334 bp. We considered length of 1500 bp and truncated the sequences longer than that.

The negative set i.e., the set of non-enhancer sequences, is generated from hg19 reference genome with similar length distribution and GC content. Set of non-enhancer sequences are generated randomly similar to positive set with matched repeat. The genNullSeqs() function from package gkmSVM^34^ in R was used to generate null sequences (negative set) with matching repeat and GC content as the input bed file for positive set regions^17^.

### Feature Extraction

To extract feature vector from positive and negative sets, we generated permutation of bases at position 1 to 6 and then calculated the frequencies of each permutation for each sequence. For other features, we generated the random walk of each sequence according to purine-pyrimidine model, and calculated statistical features and non-linear features.

The feature vector is formed by calculating k-mer with length ranging from 1 to 6 (a total of 5460 k-mer features) along with statistical and non-linear features calculated from DNA random walk^35,36^.

### Statistical Features

The basic features such as max, min, skewness, kurtosis or generic patterns such as peaks are usual measures to describe time series. In addition, we also calculated *interquartile* range, zero crossing rate, mean crossing rate, pairwise correlation and spectral entropy of each timeseries obtained from random walk.

### Nonlinear dynamic features from sequences through DNA random walk model

**Figure 2:**
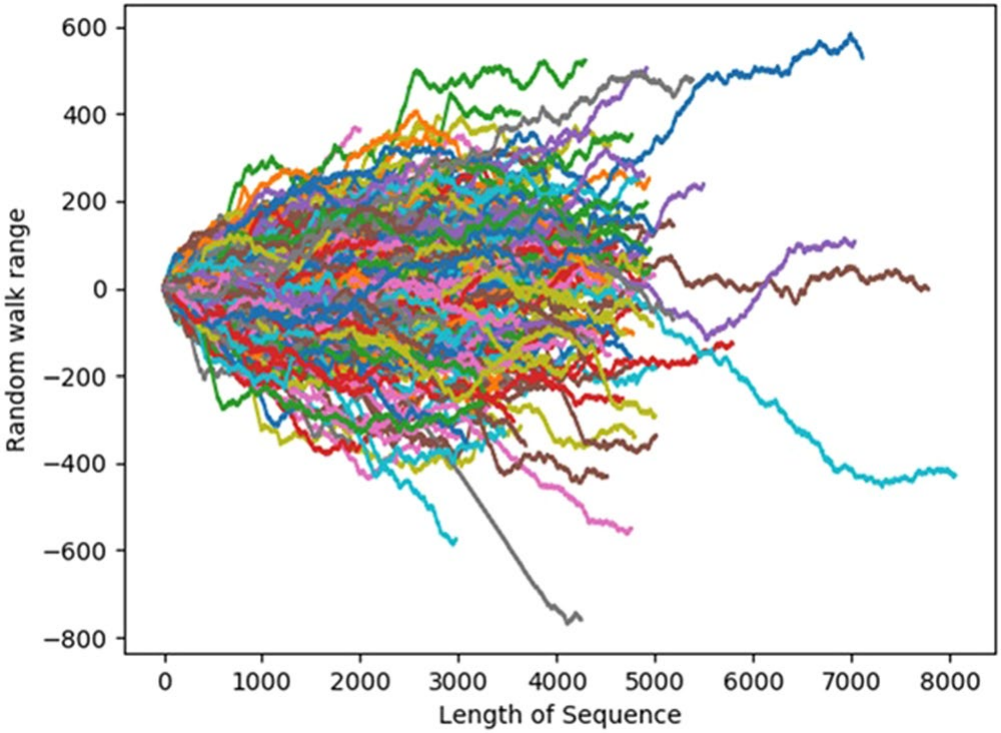
Random walk in enhancers.

In order to study dynamics of the DNA sequences, we first introduce graphical representation of DNA sequences as shown in Fig. 2, which we call as the DNA walk. For the conventional one-dimensional random walk model^35,36^, a walker moves either left [*x*(*i*) = +1] or right [*x*(*i*) = +1] a single unit length for each step *i* of the walk. For the case of an uncorrelated walk, the direction of each step is independent of the previous step. For the case of a correlated random walk, the direction of each step depends on the history (“memory”) of the walker. The DNA walk is defined by the rule that the walker steps left [*x*(*i*) = +1] if a pyrimidine occurs at position *a* linear distance *i* along the DNA chain, while the walker steps down [*x*(*i*) = −1] if a purine occurs at position *i*. The DNA walk is generated for each sequence in dataset from such a model.

Random walk is a non-stationary time series, therefore nonlinear methods are used in this study to extract features such as Hurst exponent^37^, sample entropy, Lyapunov exponent^38^ and deterrent fluctuation analysis^39^. A complete list of 5468 features (including all k-mer features) used in this paper to design enhancer prediction model can be referred in the supplementary information S1.

Sample entropy is a useful measure to investigate the dynamics of time series. It is defined as the negative natural logarithm of the conditional probability, that the subseries of length *m* remains similar at the next point, excluding self-matches. A lower value for the sample entropy therefore corresponds to a higher probability indicating more self-similarity.

The Hurst exponent is used in fractal and random walk analysis. It is used as a measure for the *long-term memory* i.e., the long statistical dependencies in the data that do not originate from cycles. It was developed by H.E. Hursts for studying the problem of long-term storage in water reservoirs.

Hurst exponent is estimated by dividing time series of full length to a number of shorter time series and rescaled range is calculated for each of the smaller time series. The rescaled range and chunk size follow a power law and the Hurst exponent is given by the exponent of this power law.

1 $$\frac{R}{\sigma }={(\frac{N}{2})}^{K}$$

In this equation, K is called the Hurst exponent. Its value is 0.5 for a purely Brownian motion, but it takes a greater value for time series that exhibits a bias in one direction.

Lyapunov exponent quantifies the exponential divergence of initially closed state trajectories and estimates the amount of chaos in the system. The dynamics of the data are reconstructed using a delay embedding method with a lag, such that each value *x*_*i*_ of the data is mapped to the vector:

2 $${X}_{i}=[{x}_{i},{x}_{i+lag},\,{x}_{i+2\ast lag},\ldots \ldots ,{x}_{i+(em{b}_{d}im-1)\ast lag}]\,$$

For each such vector *X*_*i*_, we find the closest neighbor *X*_*j*_ using the Euclidean distance. We know that, as we follow the trajectories from *X*_*i*_ and *X*_*j*_ in a chaotic system, the distances between *X*_*i*+*k*_ and *X*_*j*+*k*_ denoted as *d*_*i*_(*k*) will increase according to power law *d*_*i*_(*k*) = *c* * *e*^λ*k^, where *λ* is a good approximation of the highest Lyapunov exponent, because the exponential expansion along the axis associated with this exponent will quickly dominate the expansion or contraction along other axes.

Long range correlation in non-coding DNA sequences are observed through detrended fluctuation analysis. Detrended fluctuation analysis is used same as the Hurst component, to find long term statistical dependencies in time series. The time series is divided into windows and standard deviation of each window is calculated. The local trends are removed for each window separately by fitting a polynomial *p*_*n*_,_*i*_ to the window *W*_*n*_,_*i*_ and then calculating *W*_*n*_,_*i*_ − *p*_*n*_,_*i*_ (element-wise subtraction). The standard deviation std(X, n) is then calculated as before, only using the *detrended* window.

Enhancers are generally present in non-coding sequences of human genome where they are spread across 98% non-coding region, and this contributes towards large search space to identify enhancer sequences^19^. Number of enhancer sequences are small in comparison to sequences which do not show enhancer activity^40^. Hence, while preparing the training dataset, we maintained this ratio between positive and negative samples of the prepared dataset, with the aim to evolve a robust model. This resulted in class imbalanced training dataset created for enhancer prediction in this study and we successfully handled it using Ensemble methods.

### Classification methods for proposed model

We experimented with a number of classifiers for training the model on prepared dataset. In this section we report methods that were highly effective for training the model.

### Ensemble methods

An ensemble combines a series of *k* learned models (or *base classifiers which is decision tree for the proposed model*), *M*_1_, *M*_2_,…*M*_*k*_, with the aim of creating an improved composite classification model, M*. A given data set, *D*, is used to create *k* training sets using bootstrap sampling, *D*_1_, *D*_2_,….*D*_*k*_, where *D*_*i*_ (1 ≤ *i* ≤ *k* − 1) is used to generate classifier *M*_*i*_. The bootstrap method samples the given training tuples uniformly with replacement. That is, each time a tuple is selected, it is equally likely to be selected again and re-added to the training set. Given a test tuple to classify, it collects the class label predictions returned from the base classifiers and outputs the class in majority. The base classifiers may make mistakes, but the ensemble will misclassify test tuple only if over half of the base classifiers are in error. Ensembles yield better results when there is significant diversity among the models. The bagged classifier often has significantly greater accuracy than a single classifier derived from D, the original training data. Ensemble method will not be considerably worse as compared to single classifier and is more robust to the effects of noisy data and overfitting^41^.

RUSBoost^33^ is hybrid sampling/boosting algorithm, which uses random undersampling (RUS) that removes examples (randomly) from the majority class until the desired balance is achieved. While many data sampling techniques are designed specifically to address the class imbalance problem, boosting is a technique that can improve the performance of any weak classifier (regardless of whether the training data is imbalanced). The most common boosting algorithm is AdaBoost^42^, which iteratively builds an ensemble of models. During each iteration, example weights are modified with the goal of correctly classifying examples in the next iteration, which were incorrectly classified during the current iteration. Upon completion, all constructed models participate in a weighted vote to classify unlabelled examples. Such a technique is particularly effective at dealing with class imbalance because the minority class examples are most likely to be misclassified and therefore given higher weights in subsequent iterations.

### Convolutional Neural Network (CNN)

CNNs are very similar to Feedforward Neural Networks (FFNN) with explicit assumption that the inputs are images, by having neurons in each layer arranged in 3 dimensions, connected to a small region of the layer before it, which makes the forward function more efficient to implement and vastly reduce the count of parameters in the network. CNN is the most suitable model for image processing with first layer being convolutional layer but we modified CNN model to make it suitable for our data by selecting specific parameters. CNN is especially useful for solving problems that involve spatial or temporal dependency in the dataset whereas FFNN is a suitable choice for a dataset with independent variables. For many basic kinds of vision jobs, CNN has been shown to outperform humans^30^.

## Results and Discussion

### General statistics of selected features

In order to form the training and test datasets, the broad categories of features extracted from positive and negative sequences are given below:

-Dynamic/Statistical features (8 features)

-K-mer features (5460)

Some of the features extracted are namely: k-mer with length ranging from 1 to 6 (a total of 5460 k-mer features), standard deviation (sd), deterrent fluctuation analysis (dfa), autocorrelation with lag 200 (ac_200), autocorrelation with lag 300 (ac_300), autocorrelation with lag 100 (ac), sample entropy (sampen), hurst exponent (hurst), ratio value number to time series length (rvntsl) etc.

**Figure 3:**
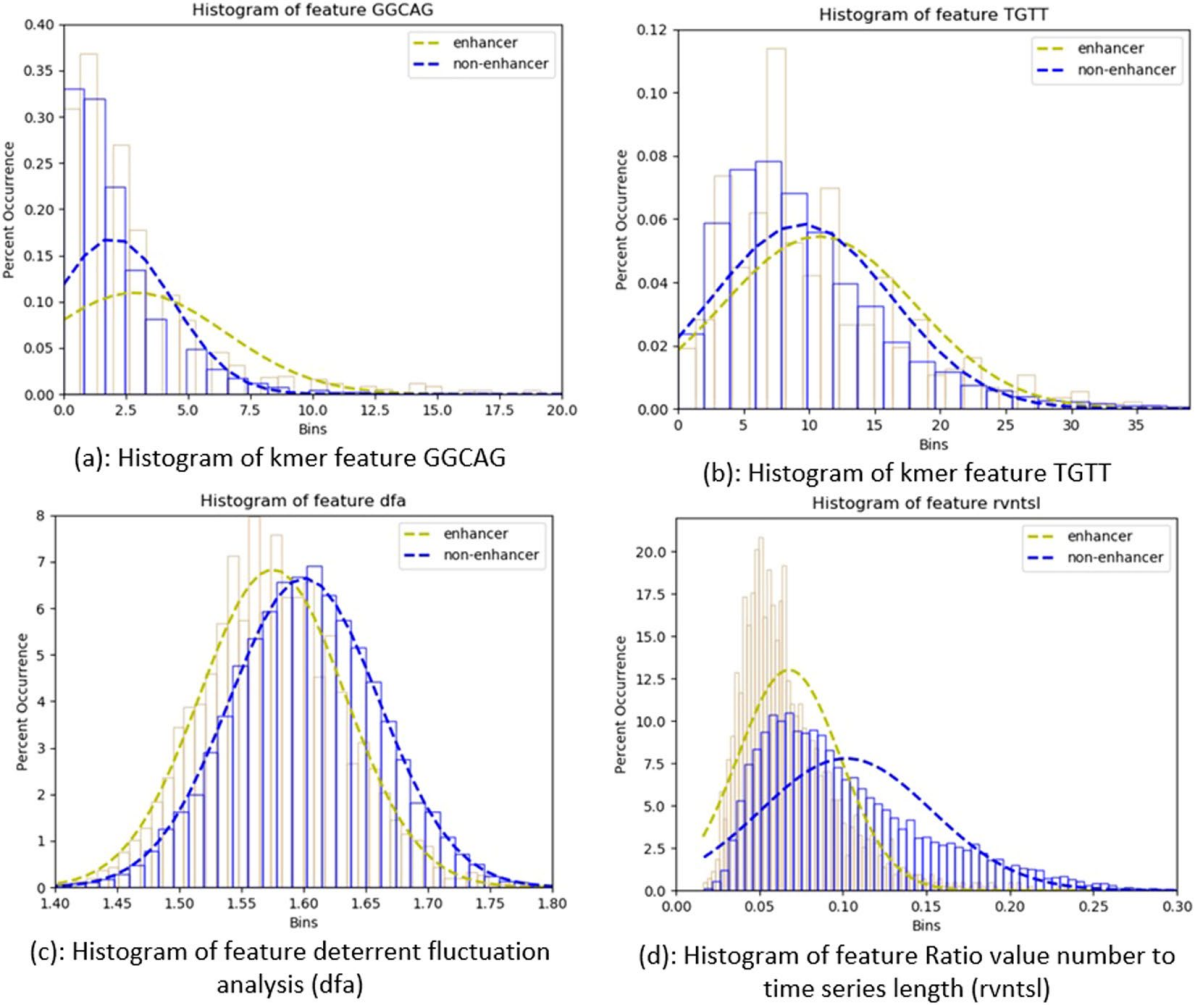
Comparison of features of training dataset using Histograms.

**Figure 4:**
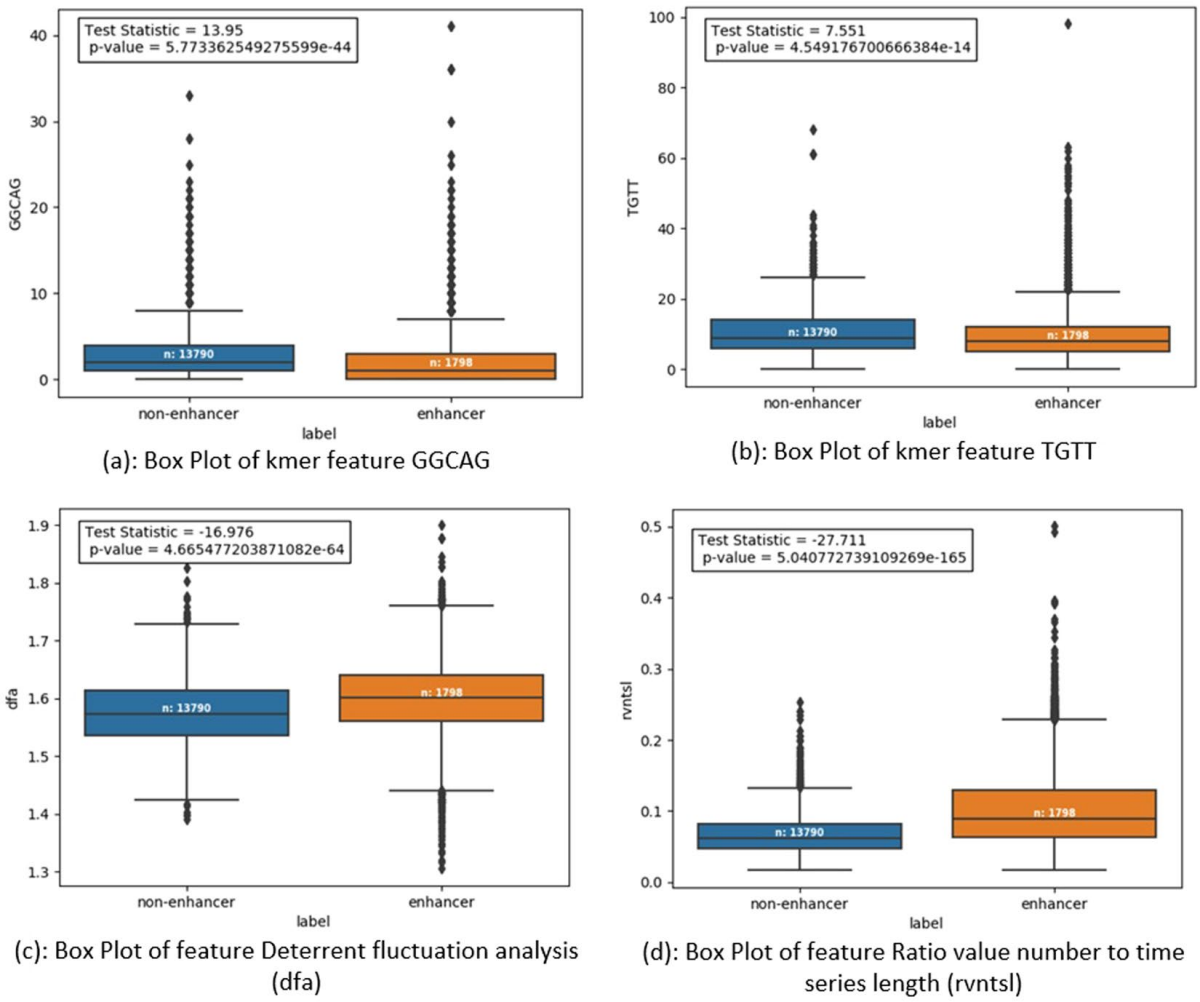
Comparison of features of training dataset using Box Plot and P-value.

Figures 3 and 4 show histograms and box plots for comparing and analysing distribution of specific features over the two class labels enhancer and non-enhancer.

Table 1 summarizes different parameters such as standard deviation (SD), mean, and p-value etc., chosen for comparison of four features namely ‘GGCAG’, ‘TGTT’, dfa, and rvntsl, against positive and negative class samples of the prepared dataset. Unpaired two tailed T-test is performed on positive and negative class samples, considering them as two different datasets to be compared. T-test is used to compare two samples to compute whether they are different from each other, and if they are different, how significantly they differ. For the four specific features chosen, p-value is much less than the value 0.05; so with great evidence, we reject the null hypothesis of identical means or high similarity of features from positive and negative samples of the prepared dataset. Therefore, we can conclude that the training data set is robust enough for successful training of a classifier to predict enhancer or non-enhancer.

**Table 1:** Comparison of features of Positive and Negative samples.

Feature name	For positive samples (i.e. enhancer)		For negative samples (i.e. non-enhancer)		Test Statistic	p-value
	SD	Mean	SD	Mean		
‘GGCAG’	3.63	2.88	2.37	1.99	13.95	5.77e-44
‘TGTT’	7.31	10.76	6.82	9.45	7.55	4.55e-14
Dfa	0.05	1.57	0.06	1.60	−16.98	4.67e-64
Rvntsl	0.03	0.07	0.05	0.10	−27.71	5.04e-165

### Experimental Design and Preparation of datasets

**Figure 5:**
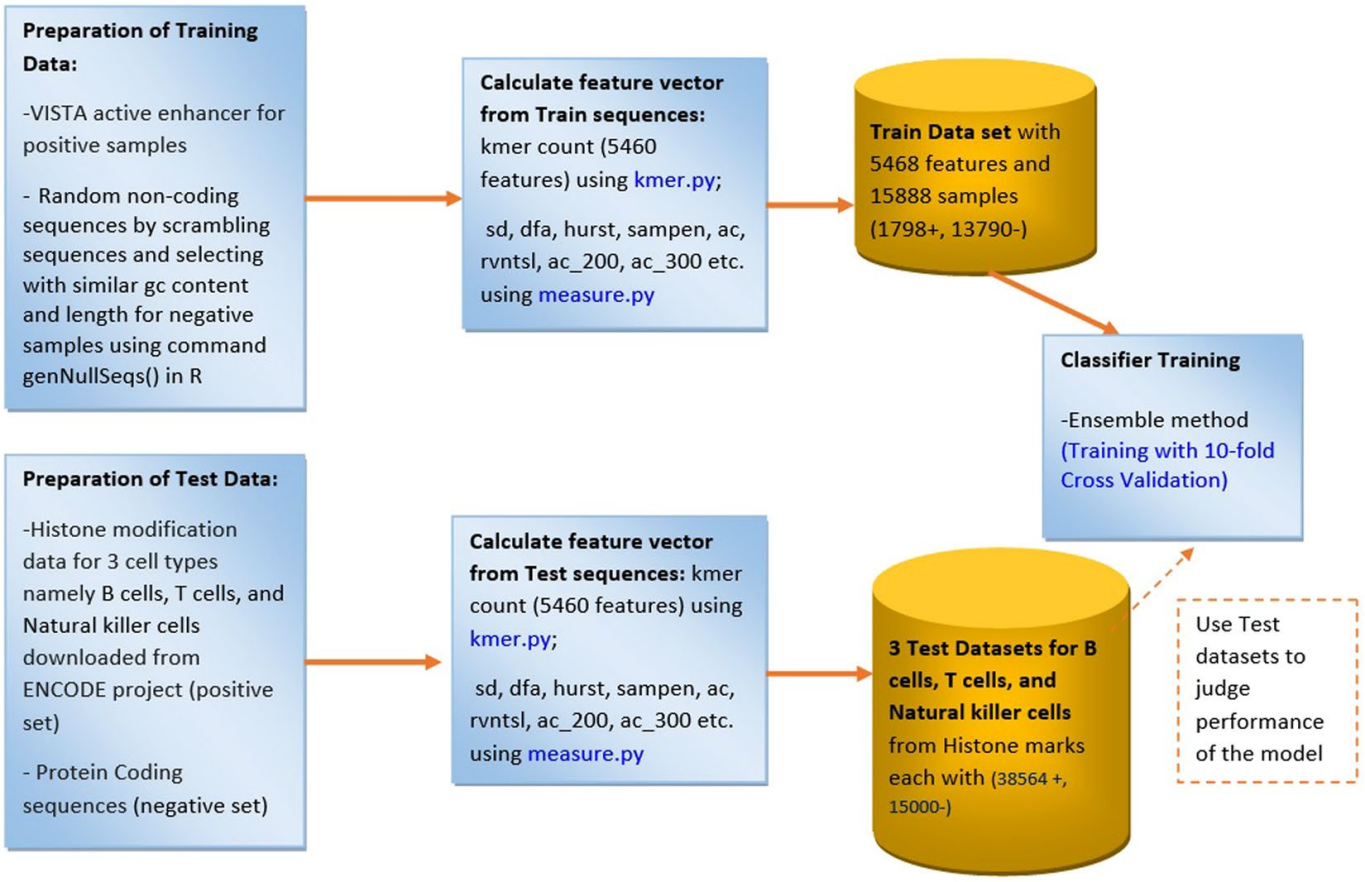
Experimental Design of Proposed System.

Figure 5 shows the experimental design of the proposed system for enhancer prediction and the following sub sections explain each part.

### Training Dataset

Training dataset is prepared by extracting k-mer features, statistical features, and nonlinear dynamic features through random walk model from positive and negative sequences, taken from VISTA Enhancer Browser. The prepared training dataset suffers from the class imbalance problem as the main class of interest (enhancer) is represented by fewer tuples as compared to the other class (non-enhancer). There are total 15,588 training samples, out of which only 1,798 are positive samples and 13,790 are negative samples. Each sample has 5,468 features in the prepared training dataset (Supplementary Dataset.csv). Ensemble methods are the most suitable methods for such class-imbalanced dataset. A test data set was formed by separating 25% of training dataset of size 15,588 samples, in order to evolve the best classifier while training of the classifiers.

### Test or Validation Datasets

In order to validate and evaluate the performance of the proposed model, we prepared three test datasets. Histone modification data for three cell types namely B cells, T cells, and Natural killer cells were downloaded from ENCODE. The bed tools with intersect and subtract command is used to extract overlapped regions. We extracted overlapped regions between H3K4me1 and H3K27ac histone modification marks as positive set (set of enhancer sequences)^29^ and protein coding sequences as negative set (set of non-enhancer sequences) for preparation of validation set to judge the performance of the proposed model.

### Training of Classifier

The foremost objective of training machine learning based model is to keep a good trade-off between simplicity of the model and the performance accuracy. The classifiers were trained on the features calculated from DNA random walk which include statistical features and non-linear features along with k-mer feature of length ranging from 1 to 6. A number of experiments were performed using classification methods such as Bagged Tree based Ensemble method^41^, RUSBoosted Tree based Ensemble method^33^, and Convolutional Neural Network (CNN)^30^, for training the model and to choose the parameters (listed in Table 2) of machine learning based methods in order to obtain the most accurate classifier. Different experimental results suggest that Ensemble learning method based on bagging is the most suitable method in comparison to other methods on the prepared training dataset.

**Table 2:** Features selected for Implementation of Computational Methods.

Bagged Tree based Ensemble method	RUSBoosted Tree based Ensemble method	CNN
Classifier: Bagged Trees Ensemble method: Bag Learner type: Decision tree Number of learners: 30 Prediction speed: ~1300 observations/sec Training Time: 348.56 secs PCA: enabled	Ensemble method: RUSBoost Learner type: Decision tree Number of learners: 30 Learning Rate: 0.1 Prediction speed: ~3000 observations/sec Training Time: 267.94 secs PCA: enabled	Input dense layer of CNN has 50 units, hidden dense layer has 10 neurons and activation function ‘tanh’, output layer has 2 neurons (1 for each classs with activation function ‘softmax’ optimizer = ‘rmsprop’ batch size = 512 Max epochs = 50 dropout = 0.25 Stopping criteria = Minimum validation error achieved with 10-fold cross validation

The choices of input parameters for implementation of different classifiers are summarized in Table 2. For the implementation of Bagged Tree based Ensemble methods, value of k i.e., number of learners, was set as 30.

As the training data has 5468 features, we used Principal Component Analysis (PCA)^43^ as a pre-processing technique before giving training dataset to ensemble methods. PCA is used to reduce a large set of variables to a small set that still contains most of the information in the large set. It basically transforms a number of (possibly) correlated variables into a (smaller) number of uncorrelated variables called principal components. The first principal component accounts for as much of the variability in the data as possible, and each succeeding component accounts for as much of the remaining variability as possible.

PCA is keeping enough components to explain 95% variance. After training, 3 components were kept with explained variance per component in order: 82.5%, 8.7%, 4.3%, 1.2%, 0.3%, 0.2%, 0.2%, 0.1%, 0.1%, 0.1%. Variance of least important components is not reported.

Another classifier that also performed comparable along with ensemble methods on the prepared training data set is Convolutional Neural Network (CNN). CNN was implemented (Keras library of Python with Tensorflow as backend) with 3 layers; namely an input dense layer, one or more hidden dense layer (s) but only one is taken for the proposed model along with an output layer. The input dense layer of CNN can have maximum of 5468 units as the same number of features are present in the prepared training dataset. The input dense layer consisted of 50 neurons and the activation function was chosen as ‘tanh’ (Hyperbolic tangent) with a dropout value of 0.25. The hidden dense layer consisted of 10 neurons with the activation function ‘tanh’ and dropout of 0.25. The dense layer is a fully connected layer that has every neuron in it connected to every neuron in the next layer. Every dense layer is using a dropout layer that is a regularization technique, which aims to reduce the complexity of the model with the goal to prevent overfitting. Dropout randomly deactivates certain units (neurons) in a layer with a certain probability p (here 25%) i.e., 25% of the activations of a layer are set to zero. Hence, the neural network doesn’t tend to rely on particular activations in any given feed-forward pass during training. As a consequence, the neural network learns different redundant representations and the network doesn’t rely on particular neurons, hence there is no dependence on combination (or interaction) of these to be present. Another advantage is that training becomes faster.

The output layer has 2 neurons (1 for each class i.e., enhancer and non-enhancer) with activation function ‘softmax’ (normalized exponential function). The model is trained on 75% of the training data with 10-fold cross validation. The rest 25% data has been used for testing the model. Training is done for maximum number of 50 epochs on the batch size of 512, with the stopping criterion being achievement of minimum validation error. It simply divides training data samples into a set of 512 samples and each set is used for training the network one by one. An epoch is completed, once all the sets are exhausted. With different batches of samples, the network trains faster, uses fewer samples at a time, and also requires lesser memory. But the batch size chosen should be optimum i.e., not too small or not too big.

For implementation of CNN method for the proposed model, the first layer chosen is dense layer and not the convolutional layer. The reason for not using the convolutional layer as first layer is that the input layer did not seem to have any positive effect on the performance of the model on test data. Multiple experiments were performed to choose these parameters of CNN model in order to obtain the most accurate model.

**Figure 6:**
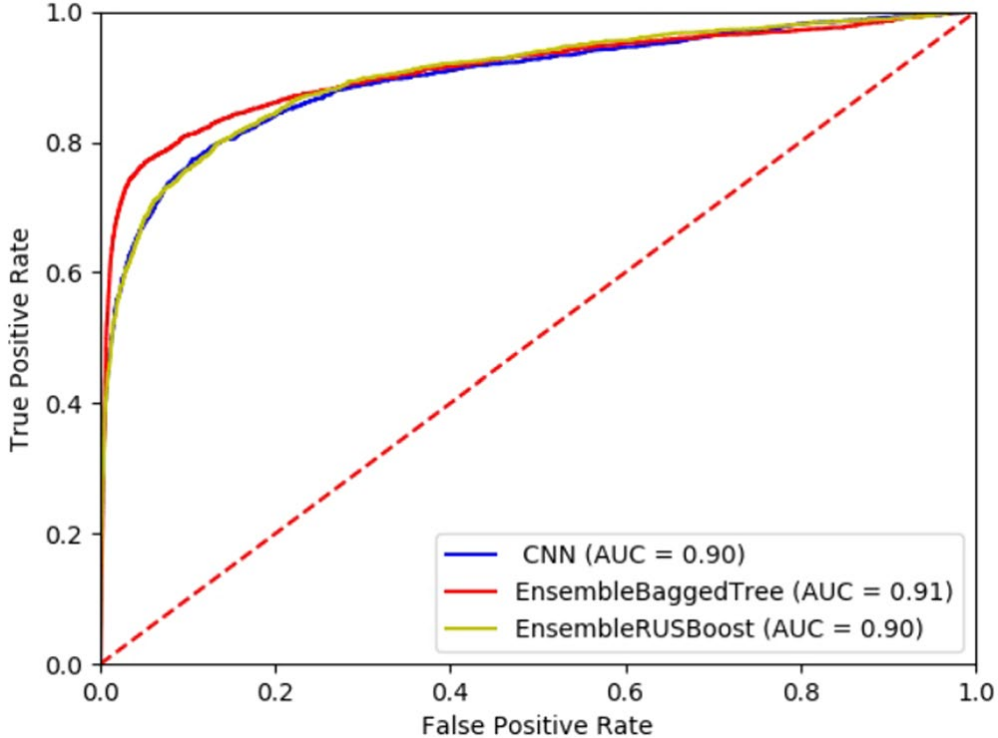
Performance of Model for different Classifiers on test data from VISTA Enhancer Browser.

To compare the performance of different classifiers for the proposed model, we plotted Receiver Operating Characteristic Curves (ROCs) on the test data formed by separating 25% of training data which is basically combination of POSITIVE and NEGATIVE enhancers as ‘gold standard enhancers’, summarized in Table 3. Additionally, the corresponding AUC was computed. Figure 6 shows performance of model for different classifiers on the prepared test data.

**Table 3:** Performance Comparison of Proposed Model with different classifiers.

Classification Methods	On true enhancer data from VISTA Enhancer Browser	
	AUC	Accuracy
Bagged Tree based Ensemble method	0.91	93.3%
RUSBoosted Tree based Ensemble method	0.90	91.3%
Convolutional Neural Network (CNN)	0.90	92.4%

**Figure 7:**
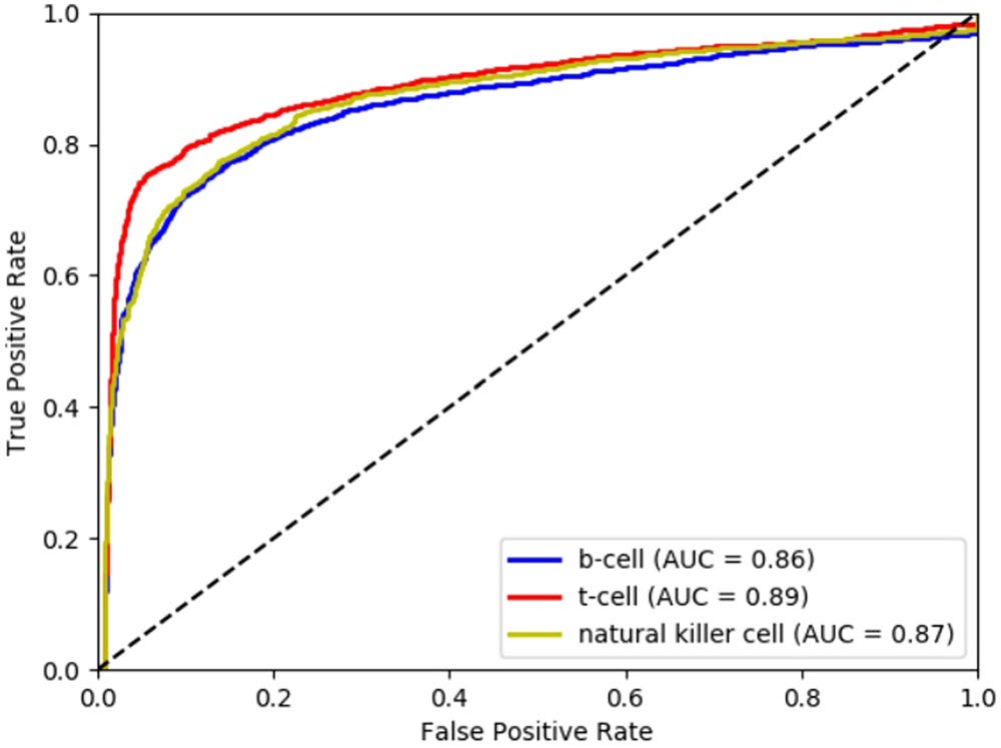
Performance of Model (Bagged Tree based Ensemble method) on Histone test data.

To assess the performance and validate the model, we used the constructed validation set to calculate AUC and plot ROCs on the prepared test datasets. We used three test datasets each with sizes of 53564 samples (38564+, 15000−) with input sequences from histone marks (for three cell types namely B cells, T cells, and Natural killer cells downloaded from ENCODE) for positive samples, and protein coding sequences for negative samples. Figure 7 shows ROCs obtained using Bagged Tree based Ensemble classifier on three test datasets of histone marks for B cells, T cells, and Natural killer cells. The results for Bagged Tree based Ensemble method and other classifiers on histone test datasets are summarized in Table 4.

**Table 4:** Performance of Proposed Model for histone test dataset.

Classification Method	B cells		T cells		Natural killer cells	
	AUC	Accuracy	AUC	Accuracy	AUC	Accuracy
Bagged Tree based Ensemble method	**0.86**	**77.8%**	**0.89**	**78.6%**	**0.87**	**78.1%**
RUSBoosted Tree based Ensemble method	0.66	66.4%	0.73	72.0%	0.67	68.3%
Convolutional Neural Network (CNN)	0.75	74.3%	0.72	73.6%	0.71	71.0%

We obtained AUC 0.86, 0.89, and 0.87 in B cells, T cells, and Natural killer cells respectively with Bagged Tree based Ensemble method, which was the best amongst three used for training the classifier of the proposed model.

### Comparison of proposed method with other enhancer prediction methods in the literature

A comparison of most closely related and recent enhancer prediction tools existing in literature of last 6 years in terms of datasets used, features, classification methods, and accuracy with the proposed method in order to bring out peculiarities and specific contribution of this work, is shown in Table 5.

**Table 5:** Performance Comparison with other Methods in literature (with different dataset used in the proposed work).

Authors	Datasets used	Features used	Method used	AUC/Accuracy (Acc)
Bu, H., Gan, Y., Wang, Y., Zhou, S., & Guan, J.^25^ EnhancerDBN^25^	Histone modification	DNA sequence compositional features, DNA methylation (GC content and DNA methylation)	Deep Belief Network	Acc 92.0%
Yang, B., Liu, F., Ren, C., Ouyang, Z., Xie, Z., Bo, X., & Shu, W.^8^ BiRen^8^	Human and mouse noncoding fragments in the VISTA Enhancer Browser	DNA sequence alone	Deep-learning-based hybrid architecture that integrates a Convolutional Neural Network (CNN) and a GRU-BRNN	AUC 0.956
Liu, F., Li, H., Ren, C., Bo, X., & Shu, W.^10^. PEDLA^10^	Histone modifications (ChIPSeq), TFs and cofactors (ChIP-Seq), chromatin accessibility (DNase-Seq), transcription (RNA-Seq), DNA methylation (RRBS), CpG islands, evolutionary conservation, sequence signatures, and occupancy of TFBS.	1,114-dimensional heterogeneous features in H1 cells, 22 training cell types/tissues	Deep learning	Acc 97.65%
Kim, S. G., Harwani, M., Grama, A., & Chaterji, S.^11^. EP-DNN^11^	Chromatin features	p300 binding sites, as enhancers, and TSS and random non-DHS sites, as non-enhancers. We perform same-cell and cross-cell predictions to quantify the validation rate and compare against two state-of-the-art methods, DEEP-ENCODE and RFECS	Deep neural network (DNN)	Acc 91.6%
Liu, B., Fang, L., Long, R., Lan, X., & Chou, K. C.^15^ iEnhancer-2L^15^	Chromatin state information of nine cell lines, including H1ES, K562, GM12878, HepG2, HUVEC, HSMM, NHLF, NHEK and HMEC	Physical structural property of inucleotide (Rise *P*_1_, Roll *P*_2_, Shift *P*_3_, Slide *P*_4_, Tilt *P*_5_, Twist *p*_6_)	SVM classification with RBF kernel function	Acc 76.89%, AUC 0.85
Kleftogiannis, D., Kalnis, P., & Bajic, V. B.^9^ DEEP^9^	Histone modification marks	Sequence characteristics	Ensemble SVM	Acc 90.2%
Rajagopal, N., Xie, W., Li, Y., Wagner, U., Wang, W., Stamatoyannopoulos, J., & Ren, B.^12^, RFECS^12^	24 Histone modifications in two distinct human cell types, embryonic stem cells and lung fibroblasts (H1 and IMR90 datasets)	p300 ENCODE data in H1 and made enhancer predictions in 12 ENCODE cell-types using the three marks H3K4me1, H3K4me3 and H3K27ac Multiple chromatin marks	Random forests	Acc 95%
Fernandez, M., & Miranda-Saavedra, D.^23^ ChromaGenSVM^23^	Histone epigenetic marks	Optimum combination of Epigenetic profiles computed at various window sizes (1, 2.5, 5, 7.5, 10, 12.5 and 15 kb).	Genetic algorithm optimized Support vector machines	Acc 85.1% AUC 0.966
Proposed method	VISTA Enhancer Browser (experimentally validated hg19)	K-mer frequency, Statistical and Non-linear features (sd, dfa, hurst, sampan, ac, rvntsl, ac_200, ac_300)	Ensemble Method (Bagged Tree)	Acc 93.3%, AUC 0.91 on test data from VISTA Enhancer Browser

Our approach basically takes experimentally validated enhancer sequences from VISTA Enhancer Browser to generate the proposed model and then validates the model on histone data as test data. We obtained accuracy of 93.3% for proposed method based on Ensemble method (Bagged Tree) with multiple k-mer and more elaborate set of features. The tool BiRen^8^ follows the similar approach for preparing their train and test datasets but they applied deep learning method^31,32^ to automatically extract the features from the sequences alone. However, the rest of the models listed in Table 5, follow an approach of taking the histone data to generate the model with different machine learning methods, and then reporting the accuracy of their model by testing it on data from VISTA Enhancer Browser. Experimental results demonstrate that the proposed method is conducive for enhancer prediction and gives new insights into role of descriptive set of features.

### Flow chart of the designed Enhancer Prediction tool

**Figure 8:**
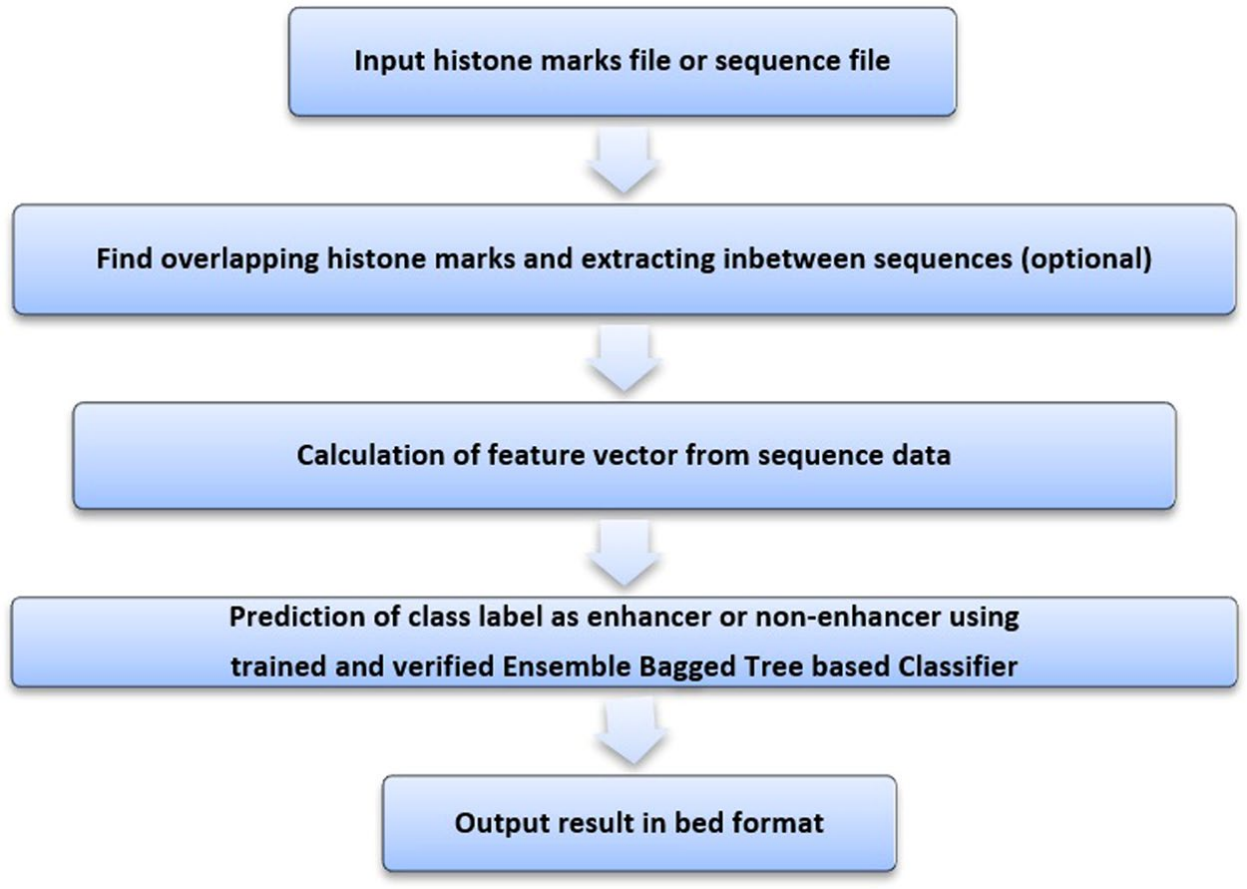
Flow graph of designed Enhancer prediction tool.

A bioinformatics tool is also developed and made available for the readers to test their reference sequences using the proposed model (Bagged Tree based Ensemble method) for classifying it as enhancer or non-enhancer. Flow graph for this tool is shown in Fig. 8. This tool is available on the following link: https://github.com/atger/enhancer_prediction.

## Conclusions

In this study, we present a model based on one-dimensional DNA random walk to precisely identify enhancer elements on a genomic scale using only the DNA sequences as input. To resolve the problem of cell specificity and requirement of high throughput dataset for enhancer prediction, the proposed method adopts time series based feature extraction using experimentally validated non-coding elements derived from the VISTA Enhancer Browser, that have gene enhancer activity, as assessed in human genome. The model integrates the nonlinear features and Ensemble method (Bagged Tree) for handling the long-range correlation in non-coding DNA to successfully resolve the challenge of the identification of enhancers using the DNA sequence. While comparing the performance of proposed model with existing methods in literature especially with the most recent ones such as BiRen^8^, PEDLA^10^, and EnhancerDB^25^, we observed that the proposed method obtained accuracy of 93.3% and AUC 0.91 on gold standard enhancer test dataset which is comparable. While assessing the performance of the proposed model on constructed validation set of histone marks, we obtained AUC 0.86, 0.89, and 0.87 in B cells, T cells, and Natural killer cells respectively. The experimental results suggest that the nonlinear dynamic features of the DNA random walk are good candidates for classifying DNA sequences into enhancer or non-enhancer. The proposed approach promises to aid in deciphering the transcriptional regulatory code located in the four-letter ‘alphabet’ of enhancer sequences in human genome.

## Electronic supplementary material

Supplementary Information

Dataset 1
