# Supplementary material for: Sequence based prediction of enhancer regions from DNA random walk
Source: Sci Rep. 2018 Oct 29;8:15912. doi: 10.1038/s41598-018-33413-y (PMC6206163; doi:10.1038/s41598-018-33413-y)
Supplement: Supplementary file 1 — Supplementary Information [file 41598_2018_33413_MOESM1_ESM.pdf]

Sequence based prediction of enhancer regions from DNA random walk

Anand Pratap Singh[1], Sarthak Mishra[1], Suraiya Jabin[1]

[1]Department of Computer Science, Jamia Millia Islamia, Jamia Nagar, New Delhi–110025, New Delhi, India. Correspondence and requests for materials should be addressed to S.J. (email: sjabin@jmi.ac.in)

Supplementary Information S1:

This supplementary information file briefly describes links for all data sources (positive and negative enhancer sequences), python scripts used for feature extraction, along with steps for creation of Training Data and Test data.

I) Sequence downloaded from VISTA Enhancer browser for human genome

Source :
https://enhancer.lbl.gov/cgi-bin/imagedb3.pl?search.org=Human&keyword=&form=searchGene&action=search&experiment=1

II) Negative Set generated using gkmSVM genNullSeqs function

Source:
https://cran.r-project.org/web/packages/gkmSVM/index.html

Example:

genNullSeqs('posSet.bed', nMaxTrials=10, xfold=8, genomeVersion='hg19', outputPosFastaFN ='pos29june.fa', outputBedFN='ctcfneg_1x.bed', outputNegFastaFN='1x29june.fa')

III) Feature vector is created using kmer.py and measure.py

Python scripts for extracting K-mer and Non-linear features are given below so that a reader can use these scripts to prepare feature vectors from sequences of his own interest.

1. Python script for extracting kmer features (kmer.py):

```
##################
# Generate K-mer feature vector
# Input: Sequence in multi fasta format
# Output: Feature vector in comma seprated file
#
# Usage (on command line prompt): python3  kmer.py  <sequence file>
e.g. ctcfneg_1x29june.fa
##################

# generate_kmer function return list of permutation possible till n position.
def generate_kmer(n):
```

```
    kmer = [ ]
    for k in range(1,n+1):
        kmer.extend([''.join(i) for i in product('ATGC',repeat=k)])
    return kmer

#  calculate frequency of each permutation in the sequence
# s : sequence
# kmer : permutations of ATGC till position n
def calculate_freq(s,kmer):
    freq = [ ]
    for i in kmer:
        freq.append(s.count(i))
    return freq

# Generating frequency matrix of kmers for each sequence and storing in dataframe.
sequence = [ ]
for record in SeqIO.parse(sys.argv[1],"fasta"):
    sequence.append(record.seq.upper())

kmer = generate_kmer(6)
df = pd.DataFrame([calculate_freq(i,kmer) for i in sequence])
df.columns = kmer

# writing output to kmer_data.csv file
df.to_csv("kmer_data.csv")
```

2. Python script for extracting Non-linear features (measure.py):

```
######################
# Generate Nonlinear feature vector
# Input : DNA Sequence in multifasta format
# Output : Non-linear feature vector in comma separated file
#
# Usage (on command line prompt): python3 measure.py <sequence file> <output file>
######################


import re
import sys
import nolds
import numpy as np
import pandas as pd
from Bio import SeqIO

# read sequences from fasta format
# s: filename
def read_seq(s):
    p = re.compile(r'>.*\n')
    d = p.split(open(s).read(),maxsplit=0)
    d = [i.replace('\n','') for i in d]
    return d[1:]
```

```
# generate walk according to purine pyrimidine model
# x : sequence
def lwalk(x):
    disp = []
    count = 0
    for i in x:
        if 'A' == i or 'G' == i:
            count += 1
        elif 'T' == i or 'C' == i:
            count -= 1
        disp = np.append(disp,count)
    return disp

# calculate autocorrelation of sequence x with some lag
def autocorrelation(x,lag):
    i = pd.Series(x)
    return pd.Series.autocorr(i,lag)

# calculate ratio value number to time series length for sequence x
def ratio_value_number_to_time_series_length(x):
    if len(x) == 0:
        return np.nan
    return len(set(x))/len(x)

# calculate all feature for each sequence in s
# s: list of sequences
def load_feature(s):
    rw = [lwalk(i) for i in s]
    sd = [np.std(i) for i in rw]
    dfa = [nolds.dfa(i) for i in rw]
    hurst = [nolds.hurst_rs(i) for i in rw]
    sampen = [nolds.sampen(i) for i in rw]
    ac = [autocorrelation(i,100) for i in rw]
    rvntsl = [ratio_value_number_to_time_series_length(i) for i in rw]
    ac_200 = [autocorrelation(i,200) for i in rw]
    ac_300 = [autocorrelation(i,300) for i in rw]
    lyapr = [nolds.lyap_r(i) for i in rw]
    inpv = pd.DataFrame([sd,dfa,hurst,sampen,ac,rvntsl,ac_200,ac_300,lyapr])
    return inpv.transpose()

# reading sequence file
sequence = [ ]
for record in SeqIO.parse(sys.argv[1],"fasta"):
    sequence.append(record.seq.upper())

# generating feature vector and saving in comma seprated file
df = load_feature(sequence)
df.columns = ['sd','dfa','hurst','sampen','ac','rvntsl','ac_200','ac_300','lyapr']
df.to_csv(sys.argv[2])
```

IV) Steps for preparation of Test Dataset:

1) Sequence Data for histone marks are downloaded from ENCODE project

2) Overlaped sequence between H3K4me1 and H3K27ac1 marks are calculated using bedtools for respective cells

3) From overlapped bed file, sequences are extracted using bedtools from hg19 genome (downladed from ucsc)

4) Kmer.py and measure.py are used to extract features from the sequences

5) Performance of model is checked on this test data (separate from training data)

Sources:

https://www.encodeproject.org/files/ENCFF044SOD/@@download/ENCFF044SOD.bed.gz

T Cell H3K27ac

https://www.encodeproject.org/files/ENCFF343VOU/@@download/ENCFF343VOU.bed.gz

Natural Killer Cell H3K4me1

https://www.encodeproject.org/files/ENCFF407DJB/@@download/ENCFF407DJB.bed.gz

Natural Killer Cell H3K27ac

https://www.encodeproject.org/files/ENCFF755QEH/@@download/ENCFF755QEH.bed.gz

B Cell H3K27ac

https://www.encodeproject.org/files/ENCFF579EPE/@@download/ENCFF579EPE.bed.gz

B Cell H3K4me1

https://www.encodeproject.org/files/ENCFF259HOW/@@download/ENCFF259HOW.bed.gz

Human Genome Hg19

http://hgdownload.cse.ucsc.edu/goldenPath/hg19/bigZips/hg19.2bit

Supplementary Information S2:

A Complete list of 5468 features extracted for the prepared Training/Test dataset:

A,T,G,C,AA,AT,AG,AC,TA,TT,TG,TC,GA,GT,GG,GC,CA,CT,CG,CC,AAA,AAT,AAG,AAC,ATA,ATT,ATG,ATC,AGA,AGT,AGG,AGC,ACA,ACT,ACG,ACC,TAA,TAT,TAG,TAC,TTA,TTT,TTG,TTC,TGA,TGT,TGG,TGC,TCA,TCT,TCG,TCC,GAA,GAT,GAG,GAC,GTA,GTT,GTG,GTC,GGA,GGT,GGG,GGC,GCA,GCT,GCG,GCC,CAA,CAT,CAG,CAC,CTA,CTT,CTG,CTC,CGA,CGT,CGG,CGC,CCA,CCT,CCG,CCC,AAAA,AAAT,AAAG,AAAC,AATA,AATT,AATG,AATC,AAGA,AAGT,AAGG,AAGC,AACA,AACT,AACG,AACC,ATAA,ATAT,ATAG,ATAC,ATTA,ATTT,ATTG,ATTC,ATGA,ATGT,ATGG,ATGC,ATCA,ATCT,ATCG,ATCC,AGAA,AGAT,AGAG,AGAC,AGTA,AGTT,AGTG,AGTC,AGGA,AGGT,AGGG,AGGC,AGCA,AGCT,AGCG,AGCC,ACAA,ACAT,ACAG,ACAC,ACTA,ACTT,ACTG,ACTC,ACGA,ACGT,ACGG,ACGC,ACCA,ACCT,ACCG,ACCC,TAAA,TAAT,TAAG,TAAC,TATA,TATT,TATG,TATC,TAGA,TAGT,TAGG,TAGC,TACA,TACT,TACG,TACC,TTAA,TTAT,TTAG,TTAC,TTTA,TTTT,TTTG,TTTC,TTGA,TTGT,TTGG,TTGC,TTCA,TTCT,TTCG,TTCC,TGAA,TGAT,TGAG,TGAC,TGTA,TGTT,TGTG,TGTC,TGGA,TGGT,TGGG,TGGC,TGCA,TGCT,TGCG,TGCC,TCAA,TCAT,TCAG,TCAC,TCTA,TCTT,TCTG,TCTC,TCGA,TCGT,TCGG,TCGC,TCCA,TCCT,TCCG,TCCC,GAAA,GAAT,GAAG,GAAC,GATA,GATT,GATG,GATC,GAGA,GAGT,GAGG,GAGC,GACA,GACT,GACG,GACC,GTAA,GTAT,GTAG,GTAC,GTTA,GTTT,GTTG,GTTC,GTGA,GTGT,GTGG,GTGC,GTCA,GTCT,GTCG,GTCC,GGAA,GGAT,GGAG,GGAC,GGTA,GGTT,GGTG,GGTC,GGGA,GGGT,GGGG,GGGC,GGCA,GGCT,GGCG,GGCC,GCAA,GCAT,GCAG,GCAC,GCTA,GCTT,GCTG,GCTC,GCGA,GCGT,GCGG,GCGC,GCCA,GCCT,GCCG,GCCC,CAAA,CAAT,CAAG,CAAC,CATA,CATT,CATG,CATC,CAGA,CAGT,CAGG,CAGC,CACA,CACT,CACG,CACC,CTAA,CTAT,CTAG,CTAC,CTTA,CTTT,CTTG,CTTC,CTGA,CTGT,CTGG,CTGC,CTCA,CTCT,CTCG,CTCC,CGAA,CGAT,CGAG,CGAC,CGTA,CGTT,CGTG,CGTC,CGGA,CGGT,CGGG,CGGC,CGCA,CGCT,CGCG,CGCC,CCAA,CCAT,CCAG,CCAC,CCTA,CCTT,CCTG,CCTC,CCGA,CCGT,CCGG,CCGC,CCCA,CCCT,CCCG,CCCC,AAAAA,AAAAT,AAAAG,AAAAC,AAATA,AAATT,AAATG,AAATC,AAAGA,AAAGT,AAAGG,AAAGC,AAACA,AAACT,AAACG,AAACC,AATAA,AATAT,AATAG,AATAC,AATTA,AATTT,AATTG,AATTC,AATGA,AATGT,AATGG,AATGC,AATCA,AATCT,AATCG,AATCC,AAGAA,AAGAT,AAGAG,AAGAC,AAGTA,AAGTT,AAGTG,AAGTC,AAGGA,AAGGT,AAGGG,AAGGC,AAGCA,AAGCT,AAGCG,AAGCC,AACAA,AACAT,AACAG,AACAC,AACTA,AACTT,AACTG,AACTC,AACGA,AACGT,AACGG,AACGC,AACCA,AACCT,AACCG,AACCC,ATAAA,ATAAT,ATAAG,ATAAC,ATATA,ATATT,ATATG,ATATC,ATAGA,ATAGT,ATAGG,ATAGC,ATACA,ATACT,ATACG,ATACC,ATTAA,ATTAT,ATTAG,ATTAC,ATTTA,ATTTT,ATTTG,ATTTC,ATTGA,ATTGT,ATTGG,ATTGC,ATTCA,ATTCT,ATTCG,ATTCC,ATGAA,ATGAT,ATGAG,ATGAC,ATGTA,ATGTT,ATGTG,ATGTC,ATGGA,ATGGT,ATGGG,ATGGC,ATGCA,ATGCT,ATGCG,ATGCC,ATCAA,ATCAT,ATCAG,ATCAC,ATCTA,ATCTT,ATCTG,ATCTC,ATCGA,ATCGT,ATCGG,ATCGC,ATCCA,ATCCT,ATCCG,ATCCC,AGAAA,AGAAT,AGAAG,AGAAC,AGATA,AGATT,AGATG,AGATC,AGAGA,AGAGT,AGAGG,AGAGC,AGACA,AGACT,AGACG,AGACC,AGTAA,AGTAT,AGTAG,AGTAC,AGTTA,AGTTT,AGTTG,AGTTC,AGTGA,AGTGT,AGTGG,AGTGC,AGTCA,AGTCT,AGTCG,AGTCC,AGGAA,AGGAT,AGGAG,AGGAC,AGGTA,AGGTT,AGGTG,AGGTC,AGGGA,AGGGT,AGGGG,AGGGC,AGGCA,AGGCT,AGGCG,AGGCC,AGCAA,AGCAT,AGCAG,AGCAC,AGCTA,AGCTT,AGCTG,AGCTC,AGCGA,AGCGT,AGCGG,AGCGC,AGCCA,AGCCT,AGCCG,AGCCC,ACAAA,ACAAT,ACAAG,ACAAC,ACATA,ACATT,ACATG,ACATC,ACAGA,ACAGT,ACAGG,ACAGC,ACACA,ACACT,ACACG,ACACC,ACTAA,ACTAT,ACTAG,ACTAC,ACTTA,ACTTT,ACTTG,ACTTC,ACTGA,ACTGT,ACTGG,ACTGC,ACTCA,ACTCT,ACTCG,ACTCC,ACGAA,ACGAT,ACGAG,ACGAC,ACGTA,ACGTT,ACGTG,ACGTC,ACGGA,ACGGT,ACGGG,ACGGC,ACGCA,ACGCT,ACGCG,ACGCC,ACCAA,ACCAT

,ACCAG,ACCAC,ACCTA,ACCTT,ACCTG,ACCTC,ACCGA,ACCGT,ACCGG,ACCGC,ACCC
A,ACCCT,ACCCG,ACCCC,TAAAA,TAAAT,TAAAG,TAAAC,TAATA,TAATT,TAATG,TAA
TC,TAAGA,TAAGT,TAAGG,TAAGC,TAACA,TAACT,TAACG,TAACC,TATAA,TATAT,TAT
AG,TATAC,TATTA,TATTT,TATTG,TATTC,TATGA,TATGT,TATGG,TATGC,TATCA,TATC
T,TATCG,TATCC,TAGAA,TAGAT,TAGAG,TAGAC,TAGTA,TAGTT,TAGTG,TAGTC,TAGG
A,TAGGT,TAGGG,TAGGC,TAGCA,TAGCT,TAGCG,TAGCC,TACAA,TACAT,TACAG,TAC
AC,TACTA,TACTT,TACTG,TACTC,TACGA,TACGT,TACGG,TACGC,TACCA,TACCT,TAC
CG,TACCC,TTAAA,TTAAT,TTAAG,TTAAC,TTATA,TTATT,TTATG,TTATC,TTAGA,TTAG
T,TTAGG,TTAGC,TTACA,TTACT,TTACG,TTACC,TTTAA,TTTAT,TTTAG,TTTAC,TTTTA,
TTTTT,TTTTG,TTTTC,TTTGA,TTTGT,TTTGG,TTTGC,TTTCA,TTTCT,TTTCG,TTTCC,TTG
AA,TTGAT,TTGAG,TTGAC,TTGTA,TTGTT,TTGTG,TTGTC,TTGGA,TTGGT,TTGGG,TTGG
C,TTGCA,TTGCT,TTGCG,TTGCC,TTCAA,TTCAT,TTCAG,TTCAC,TTCTA,TTCTT,TTCTG,
TTCTC,TTCGA,TTCGT,TTCGG,TTCGC,TTCCA,TTCCT,TTCCG,TTCCC,TGAAA,TGAAT,T
GAAG,TGAAC,TGATA,TGATT,TGATG,TGATC,TGAGA,TGAGT,TGAGG,TGAGC,TGACA,
TGACT,TGACG,TGACC,TGTAA,TGTAT,TGTAG,TGTAC,TGTTA,TGTTT,TGTTG,TGTTC,T
GTGA,TGTGT,TGTGG,TGTGC,TGTCA,TGTCT,TGTCG,TGTCC,TGGAA,TGGAT,TGGAG,T
GGAC,TGGTA,TGGTT,TGGTG,TGGTC,TGGGA,TGGGT,TGGGG,TGGGC,TGGCA,TGGCT,
TGGCG,TGGCC,TGCAA,TGCAT,TGCAG,TGCAC,TGCTA,TGCTT,TGCTG,TGCTC,TGCGA,
TGCGT,TGCGG,TGCGC,TGCCA,TGCCT,TGCCG,TGCCC,TCAAA,TCAAT,TCAAG,TCAAC,
TCATA,TCATT,TCATG,TCATC,TCAGA,TCAGT,TCAGG,TCAGC,TCACA,TCACT,TCACG,
TCACC,TCTAA,TCTAT,TCTAG,TCTAC,TCTTA,TCTTT,TCTTG,TCTTC,TCTGA,TCTGT,TC
TGG,TCTGC,TCTCA,TCTCT,TCTCG,TCTCC,TCGAA,TCGAT,TCGAG,TCGAC,TCGTA,TCG
TT,TCGTG,TCGTC,TCGGA,TCGGT,TCGGG,TCGGC,TCGCA,TCGCT,TCGCG,TCGCC,TCC
AA,TCCAT,TCCAG,TCCAC,TCCTA,TCCTT,TCCTG,TCCTC,TCCGA,TCCGT,TCCGG,TCCG
C,TCCCA,TCCCT,TCCCG,TCCCC,GAAAA,GAAAT,GAAAG,GAAAC,GAATA,GAATT,GAA
TG,GAATC,GAAGA,GAAGT,GAAGG,GAAGC,GAACA,GAACT,GAACG,GAACC,GATAA,G
ATAT,GATAG,GATAC,GATTA,GATTT,GATTG,GATTC,GATGA,GATGT,GATGG,GATGC,
GATCA,GATCT,GATCG,GATCC,GAGAA,GAGAT,GAGAG,GAGAC,GAGTA,GAGTT,GAGT
G,GAGTC,GAGGA,GAGGT,GAGGG,GAGGC,GAGCA,GAGCT,GAGCG,GAGCC,GACAA,G
ACAT,GACAG,GACAC,GACTA,GACTT,GACTG,GACTC,GACGA,GACGT,GACGG,GACGC
,GACCA,GACCT,GACCG,GACCC,GTAAA,GTAAT,GTAAG,GTAAC,GTATA,GTATT,GTAT
G,GTATC,GTAGA,GTAGT,GTAGG,GTAGC,GTACA,GTACT,GTACG,GTACC,GTTAA,GTT
AT,GTTAG,GTTAC,GTTTA,GTTTT,GTTTG,GTTTC,GTTGA,GTTGT,GTTGG,GTTGC,GTTC
A,GTTCT,GTTCG,GTTCC,GTGAA,GTGAT,GTGAG,GTGAC,GTGTA,GTGTT,GTGTG,GTGT
C,GTGGA,GTGGT,GTGGG,GTGGC,GTGCA,GTGCT,GTGCG,GTGCC,GTCAA,GTCAT,GTC
AG,GTCAC,GTCTA,GTCTT,GTCTG,GTCTC,GTCGA,GTCGT,GTCGG,GTCGC,GTCCA,GTC
CT,GTCCG,GTCCC,GGAAA,GGAAT,GGAAG,GGAAC,GGATA,GGATT,GGATG,GGATC,G
GAGA,GGAGT,GGAGG,GGAGC,GGACA,GGACT,GGACG,GGACC,GGTAA,GGTAT,GGTA
G,GGTAC,GGTTA,GGTTT,GGTTG,GGTTC,GGTGA,GGTGT,GGTGG,GGTGC,GGTCA,GGT
CT,GGTCG,GGTCC,GGGAA,GGGAT,GGGAG,GGGAC,GGGTA,GGGTT,GGGTG,GGGTC,G
GGGA,GGGGT,GGGGG,GGGGC,GGGCA,GGGCT,GGGCG,GGGCC,GGCAA,GGCAT,GGCA
G,GGCAC,GGCTA,GGCTT,GGCTG,GGCTC,GGCGA,GGCGT,GGCGG,GGCGC,GGCCA,GG
CCT,GGCCG,GGCCC,GCAAA,GCAAT,GCAAG,GCAAC,GCATA,GCATT,GCATG,GCATC,G
CAGA,GCAGT,GCAGG,GCAGC,GCACA,GCACT,GCACG,GCACC,GCTAA,GCTAT,GCTAG,
GCTAC,GCTTA,GCTTT,GCTTG,GCTTC,GCTGA,GCTGT,GCTGG,GCTGC,GCTCA,GCTCT,
GCTCG,GCTCC,GCGAA,GCGAT,GCGAG,GCGAC,GCGTA,GCGTT,GCGTG,GCGTC,GCGG
A,GCGGT,GCGGG,GCGGC,GCGCA,GCGCT,GCGCG,GCGCC,GCCAA,GCCAT,GCCAG,GC
CAC,GCCTA,GCCTT,GCCTG,GCCTC,GCCGA,GCCGT,GCCGG,GCCGC,GCCCA,GCCCT,G
CCCG,GCCCC,CAAAA,CAAAT,CAAAG,CAAAC,CAATA,CAATT,CAATG,CAATC,CAAGA,
CAAGT,CAAGG,CAAGC,CAACA,CAACT,CAACG,CAACC,CATAA,CATAT,CATAG,CATA
C,CATTA,CATTT,CATTG,CATTC,CATGA,CATGT,CATGG,CATGC,CATCA,CATCT,CATC
G,CATCC,CAGAA,CAGAT,CAGAG,CAGAC,CAGTA,CAGTT,CAGTG,CAGTC,CAGGA,CAG

GT,CAGGG,CAGGC,CAGCA,CAGCT,CAGCG,CAGCC,CACAA,CACAT,CACAG,CACAC,C
ACTA,CACTT,CACTG,CACTC,CACGA,CACGT,CACGG,CACGC,CACCA,CACCT,CACCG,
CACCC,CTAAA,CTAAT,CTAAG,CTAAC,CTATA,CTATT,CTATG,CTATC,CTAGA,CTAGT,
CTAGG,CTAGC,CTACA,CTACT,CTACG,CTACC,CTTAA,CTTAT,CTTAG,CTTAC,CTTTA,C
TTTT,CTTTG,CTTTC,CTTGA,CTTGT,CTTGG,CTTGC,CTTCA,CTTCT,CTTCG,CTTCC,CTG
AA,CTGAT,CTGAG,CTGAC,CTGTA,CTGTT,CTGTG,CTGTC,CTGGA,CTGGT,CTGGG,CTG
GC,CTGCA,CTGCT,CTGCG,CTGCC,CTCAA,CTCAT,CTCAG,CTCAC,CTCTA,CTCTT,CTCT
G,CTCTC,CTCGA,CTCGT,CTCGG,CTCGC,CTCCA,CTCCT,CTCCG,CTCCC,CGAAA,CGAA
T,CGAAG,CGAAC,CGATA,CGATT,CGATG,CGATC,CGAGA,CGAGT,CGAGG,CGAGC,CG
ACA,CGACT,CGACG,CGACC,CGTAA,CGTAT,CGTAG,CGTAC,CGTTA,CGTTT,CGTTG,CG
TTC,CGTGA,CGTGT,CGTGG,CGTGC,CGTCA,CGTCT,CGTCG,CGTCC,CGGAA,CGGAT,CG
GAG,CGGAC,CGGTA,CGGTT,CGGTG,CGGTC,CGGGA,CGGGT,CGGGG,CGGGC,CGGCA,
CGGCT,CGGCG,CGGCC,CGCAA,CGCAT,CGCAG,CGCAC,CGCTA,CGCTT,CGCTG,CGCTC
,CGCGA,CGCGT,CGCGG,CGCGC,CGCCA,CGCCT,CGCCG,CGCCC,CCAAA,CCAAT,CCAA
G,CCAAC,CCATA,CCATT,CCATG,CCATC,CCAGA,CCAGT,CCAGG,CCAGC,CCACA,CCA
CT,CCACG,CCACC,CCTAA,CCTAT,CCTAG,CCTAC,CCTTA,CCTTT,CCTTG,CCTTC,CCTG
A,CCTGT,CCTGG,CCTGC,CCTCA,CCTCT,CCTCG,CCTCC,CCGAA,CCGAT,CCGAG,CCGA
C,CCGTA,CCGTT,CCGTG,CCGTC,CCGGA,CCGGT,CCGGG,CCGGC,CCGCA,CCGCT,CCGC
G,CCGCC,CCCAA,CCCAT,CCCAG,CCCAC,CCCTA,CCCTT,CCCTG,CCCTC,CCCGA,CCCG
T,CCCGG,CCCGC,CCCCA,CCCCT,CCCCG,CCCCC,AAAAAA,AAAAAT,AAAAAG,AAAAA
C,AAAATA,AAAATT,AAAATG,AAAATC,AAAAGA,AAAAGT,AAAAGG,AAAAGC,AAAA
CA,AAAACT,AAAACG,AAAACC,AAATAA,AAATAT,AAATAG,AAATAC,AAATTA,AAAT
TT,AAATTG,AAATTC,AAATGA,AAATGT,AAATGG,AAATGC,AAATCA,AAATCT,AAATC
G,AAATCC,AAAGAA,AAAGAT,AAAGAG,AAAGAC,AAAGTA,AAAGTT,AAAGTG,AAAG
TC,AAAGGA,AAAGGT,AAAGGG,AAAGGC,AAAGCA,AAAGCT,AAAGCG,AAAGCC,AAA
CAA,AAACAT,AAACAG,AAACAC,AAACTA,AAACTT,AAACTG,AAACTC,AAACGA,AAA
CGT,AAACGG,AAACGC,AAACCA,AAACCT,AAACCG,AAACCC,AATAAA,AATAAT,AAT
AAG,AATAAC,AATATA,AATATT,AATATG,AATATC,AATAGA,AATAGT,AATAGG,AAT
AGC,AATACA,AATACT,AATACG,AATACC,AATTAA,AATTAT,AATTAG,AATTAC,AATT
TA,AATTTT,AATTTG,AATTTC,AATTGA,AATTGT,AATTGG,AATTGC,AATTCA,AATTCT,
AATTCG,AATTCC,AATGAA,AATGAT,AATGAG,AATGAC,AATGTA,AATGTT,AATGTG,A
ATGTC,AATGGA,AATGGT,AATGGG,AATGGC,AATGCA,AATGCT,AATGCG,AATGCC,A
ATCAA,AATCAT,AATCAG,AATCAC,AATCTA,AATCTT,AATCTG,AATCTC,AATCGA,AA
TCGT,AATCGG,AATCGC,AATCCA,AATCCT,AATCCG,AATCCC,AAGAAA,AAGAAT,AAG
AAG,AAGAAC,AAGATA,AAGATT,AAGATG,AAGATC,AAGAGA,AAGAGT,AAGAGG,AA
GAGC,AAGACA,AAGACT,AAGACG,AAGACC,AAGTAA,AAGTAT,AAGTAG,AAGTAC,A
AGTTA,AAGTTT,AAGTTG,AAGTTC,AAGTGA,AAGTGT,AAGTGG,AAGTGC,AAGTCA,AA
GTCT,AAGTCG,AAGTCC,AAGGAA,AAGGAT,AAGGAG,AAGGAC,AAGGTA,AAGGTT,AA
GGTG,AAGGTC,AAGGGA,AAGGGT,AAGGGG,AAGGGC,AAGGCA,AAGGCT,AAGGCG,A
AGGCC,AAGCAA,AAGCAT,AAGCAG,AAGCAC,AAGCTA,AAGCTT,AAGCTG,AAGCTC,A
AGCGA,AAGCGT,AAGCGG,AAGCGC,AAGCCA,AAGCCT,AAGCCG,AAGCCC,AACAAA,
AACAAT,AACAAG,AACAAC,AACATA,AACATT,AACATG,AACATC,AACAGA,AACAGT,
AACAGG,AACAGC,AACACA,AACACT,AACACG,AACACC,AACTAA,AACTAT,AACTAG,
AACTAC,AACTTA,AACTTT,AACTTG,AACTTC,AACTGA,AACTGT,AACTGG,AACTGC,A
ACTCA,AACTCT,AACTCG,AACTCC,AACGAA,AACGAT,AACGAG,AACGAC,AACGTA,A
ACGTT,AACGTG,AACGTC,AACGGA,AACGGT,AACGGG,AACGGC,AACGCA,AACGCT,A
ACGCG,AACGCC,AACCAA,AACCAT,AACCAG,AACCAC,AACCTA,AACCTT,AACCTG,A
ACCTC,AACCGA,AACCGT,AACCGG,AACCGC,AACCCA,AACCCT,AACCCG,AACCCC,AT
AAAA,ATAAAT,ATAAAG,ATAAAC,ATAATA,ATAATT,ATAATG,ATAATC,ATAAGA,AT
AAGT,ATAAGG,ATAAGC,ATAACA,ATAACT,ATAACG,ATAACC,ATATAA,ATATAT,ATA
TAG,ATATAC,ATATTA,ATATTT,ATATTG,ATATTC,ATATGA,ATATGT,ATATGG,ATATG
C,ATATCA,ATATCT,ATATCG,ATATCC,ATAGAA,ATAGAT,ATAGAG,ATAGAC,ATAGTA,

ATAGTT,ATAGTG,ATAGTC,ATAGGA,ATAGGT,ATAGGG,ATAGGC,ATAGCA,ATAGCT,A TAGCG,ATAGCC,ATACAA,ATACAT,ATACAG,ATACAC,ATACTA,ATACTT,ATACTG,AT ACTC,ATACGA,ATACGT,ATACGG,ATACGC,ATACCA,ATACCT,ATACCG,ATACCC,ATT AAA,ATTAAT,ATTAAG,ATTAAC,ATTATA,ATTATT,ATTATG,ATTATC,ATTAGA,ATTAG T,ATTAGG,ATTAGC,ATTACA,ATTACT,ATTACG,ATTACC,ATTTAA,ATTTAT,ATTTAG,A TTTAC,ATTTTA,ATTTTT,ATTTTG,ATTTTC,ATTTGA,ATTTGT,ATTTGG,ATTTGC,ATTTC A,ATTTCT,ATTTCG,ATTTCC,ATTGAA,ATTGAT,ATTGAG,ATTGAC,ATTGTA,ATTGTT,A TTGTG,ATTGTC,ATTGGA,ATTGGT,ATTGGG,ATTGGC,ATTGCA,ATTGCT,ATTGCG,ATT GCC,ATTCAA,ATTCAT,ATTCAG,ATTCAC,ATTCTA,ATTCTT,ATTCTG,ATTCTC,ATTCGA ,ATTCGT,ATTCGG,ATTCGC,ATTCCA,ATTCCT,ATTCCG,ATTCCC,ATGAAA,ATGAAT,AT GAAG,ATGAAC,ATGATA,ATGATT,ATGATG,ATGATC,ATGAGA,ATGAGT,ATGAGG,AT GAGC,ATGACA,ATGACT,ATGACG,ATGACC,ATGTAA,ATGTAT,ATGTAG,ATGTAC,ATG TTA,ATGTTT,ATGTTG,ATGTTC,ATGTGA,ATGTGT,ATGTGG,ATGTGC,ATGTCA,ATGTC T,ATGTCG,ATGTCC,ATGGAA,ATGGAT,ATGGAG,ATGGAC,ATGGTA,ATGGTT,ATGGTG, ATGGTC,ATGGGA,ATGGGT,ATGGGG,ATGGGC,ATGGCA,ATGGCT,ATGGCG,ATGGCC, ATGCAA,ATGCAT,ATGCAG,ATGCAC,ATGCTA,ATGCTT,ATGCTG,ATGCTC,ATGCGA,A TGCGT,ATGCGG,ATGCGC,ATGCCA,ATGCCT,ATGCCG,ATGCCC,ATCAAA,ATCAAT,AT CAAG,ATCAAC,ATCATA,ATCATT,ATCATG,ATCATC,ATCAGA,ATCAGT,ATCAGG,ATC AGC,ATCACA,ATCACT,ATCACG,ATCACC,ATCTAA,ATCTAT,ATCTAG,ATCTAC,ATCTT A,ATCTTT,ATCTTG,ATCTTC,ATCTGA,ATCTGT,ATCTGG,ATCTGC,ATCTCA,ATCTCT,A TCTCG,ATCTCC,ATCGAA,ATCGAT,ATCGAG,ATCGAC,ATCGTA,ATCGTT,ATCGTG,ATC GTC,ATCGGA,ATCGGT,ATCGGG,ATCGGC,ATCGCA,ATCGCT,ATCGCG,ATCGCC,ATCC AA,ATCCAT,ATCCAG,ATCCAC,ATCCTA,ATCCTT,ATCCTG,ATCCTC,ATCCGA,ATCCGT, ATCCGG,ATCCGC,ATCCCA,ATCCCT,ATCCCG,ATCCCC,AGAAAA,AGAAAT,AGAAAG,A GAAAC,AGAATA,AGAATT,AGAATG,AGAATC,AGAAGA,AGAAGT,AGAAGG,AGAAGC, AGAACA,AGAACT,AGAACG,AGAACC,AGATAA,AGATAT,AGATAG,AGATAC,AGATTA, AGATTT,AGATTG,AGATTC,AGATGA,AGATGT,AGATGG,AGATGC,AGATCA,AGATCT,A GATCG,AGATCC,AGAGAA,AGAGAT,AGAGAG,AGAGAC,AGAGTA,AGAGTT,AGAGTG, AGAGTC,AGAGGA,AGAGGT,AGAGGG,AGAGGC,AGAGCA,AGAGCT,AGAGCG,AGAGC C,AGACAA,AGACAT,AGACAG,AGACAC,AGACTA,AGACTT,AGACTG,AGACTC,AGACG A,AGACGT,AGACGG,AGACGC,AGACCA,AGACCT,AGACCG,AGACCC,AGTAAA,AGTAA T,AGTAAG,AGTAAC,AGTATA,AGTATT,AGTATG,AGTATC,AGTAGA,AGTAGT,AGTAGG ,AGTAGC,AGTACA,AGTACT,AGTACG,AGTACC,AGTTAA,AGTTAT,AGTTAG,AGTTAC, AGTTTA,AGTTTT,AGTTTG,AGTTTC,AGTTGA,AGTTGT,AGTTGG,AGTTGC,AGTTCA,AG TTCT,AGTTCG,AGTTCC,AGTGAA,AGTGAT,AGTGAG,AGTGAC,AGTGTA,AGTGTT,AGT GTG,AGTGTC,AGTGGA,AGTGGT,AGTGGG,AGTGGC,AGTGCA,AGTGCT,AGTGCG,AGT GCC,AGTCAA,AGTCAT,AGTCAG,AGTCAC,AGTCTA,AGTCTT,AGTCTG,AGTCTC,AGTC GA,AGTCGT,AGTCGG,AGTCGC,AGTCCA,AGTCCT,AGTCCG,AGTCCC,AGGAAA,AGGA AT,AGGAAG,AGGAAC,AGGATA,AGGATT,AGGATG,AGGATC,AGGAGA,AGGAGT,AGG AGG,AGGAGC,AGGACA,AGGACT,AGGACG,AGGACC,AGGTAA,AGGTAT,AGGTAG,AG GTAC,AGGTTA,AGGTTT,AGGTTG,AGGTTC,AGGTGA,AGGTGT,AGGTGG,AGGTGC,AGG TCA,AGGTCT,AGGTCG,AGGTCC,AGGGAA,AGGGAT,AGGGAG,AGGGAC,AGGGTA,AGG GTT,AGGGTG,AGGGTC,AGGGGA,AGGGGT,AGGGGG,AGGGGC,AGGGCA,AGGGCT,AG GGCG,AGGGCC,AGGCAA,AGGCAT,AGGCAG,AGGCAC,AGGCTA,AGGCTT,AGGCTG,AG GCTC,AGGCGA,AGGCGT,AGGCGG,AGGCGC,AGGCCA,AGGCCT,AGGCCG,AGGCCC,AG CAAA,AGCAAT,AGCAAG,AGCAAC,AGCATA,AGCATT,AGCATG,AGCATC,AGCAGA,AG CAGT,AGCAGG,AGCAGC,AGCACA,AGCACT,AGCACG,AGCACC,AGCTAA,AGCTAT,AG CTAG,AGCTAC,AGCTTA,AGCTTT,AGCTTG,AGCTTC,AGCTGA,AGCTGT,AGCTGG,AGC TGC,AGCTCA,AGCTCT,AGCTCG,AGCTCC,AGCGAA,AGCGAT,AGCGAG,AGCGAC,AGC GTA,AGCGTT,AGCGTG,AGCGTC,AGCGGA,AGCGGT,AGCGGG,AGCGGC,AGCGCA,AGC GCT,AGCGCG,AGCGCC,AGCCAA,AGCCAT,AGCCAG,AGCCAC,AGCCTA,AGCCTT,AGC CTG,AGCCTC,AGCCGA,AGCCGT,AGCCGG,AGCCGC,AGCCCA,AGCCCT,AGCCCG,AGC

CCC,ACAAAA,ACAAAT,ACAAAG,ACAAAC,ACAATA,ACAATT,ACAATG,ACAATC,ACA
AGA,ACAAGT,ACAAGG,ACAAGC,ACAACA,ACAACT,ACAACG,ACAACC,ACATAA,ACA
TAT,ACATAG,ACATAC,ACATTA,ACATTT,ACATTG,ACATTC,ACATGA,ACATGT,ACAT
GG,ACATGC,ACATCA,ACATCT,ACATCG,ACATCC,ACAGAA,ACAGAT,ACAGAG,ACAG
AC,ACAGTA,ACAGTT,ACAGTG,ACAGTC,ACAGGA,ACAGGT,ACAGGG,ACAGGC,ACAG
CA,ACAGCT,ACAGCG,ACAGCC,ACACAA,ACACAT,ACACAG,ACACAC,ACACTA,ACAC
TT,ACACTG,ACACTC,ACACGA,ACACGT,ACACGG,ACACGC,ACACCA,ACACCT,ACACC
G,ACACCC,ACTAAA,ACTAAT,ACTAAG,ACTAAC,ACTATA,ACTATT,ACTATG,ACTATC,
ACTAGA,ACTAGT,ACTAGG,ACTAGC,ACTACA,ACTACT,ACTACG,ACTACC,ACTTAA,A
CTTAT,ACTTAG,ACTTAC,ACTTTA,ACTTTT,ACTTTG,ACTTTC,ACTTGA,ACTTGT,ACTT
GG,ACTTGC,ACTTCA,ACTTCT,ACTTCG,ACTTCC,ACTGAA,ACTGAT,ACTGAG,ACTGAC
,ACTGTA,ACTGTT,ACTGTG,ACTGTC,ACTGGA,ACTGGT,ACTGGG,ACTGGC,ACTGCA,A
CTGCT,ACTGCG,ACTGCC,ACTCAA,ACTCAT,ACTCAG,ACTCAC,ACTCTA,ACTCTT,ACT
CTG,ACTCTC,ACTCGA,ACTCGT,ACTCGG,ACTCGC,ACTCCA,ACTCCT,ACTCCG,ACTCC
C,ACGAAA,ACGAAT,ACGAAG,ACGAAC,ACGATA,ACGATT,ACGATG,ACGATC,ACGAG
A,ACGAGT,ACGAGG,ACGAGC,ACGACA,ACGACT,ACGACG,ACGACC,ACGTAA,ACGTA
T,ACGTAG,ACGTAC,ACGTTA,ACGTTT,ACGTTG,ACGTTC,ACGTGA,ACGTGT,ACGTGG,
ACGTGC,ACGTCA,ACGTCT,ACGTCG,ACGTCC,ACGGAA,ACGGAT,ACGGAG,ACGGAC,
ACGGTA,ACGGTT,ACGGTG,ACGGTC,ACGGGA,ACGGGT,ACGGGG,ACGGGC,ACGGCA,
ACGGCT,ACGGCG,ACGGCC,ACGCAA,ACGCAT,ACGCAG,ACGCAC,ACGCTA,ACGCTT,
ACGCTG,ACGCTC,ACGCGA,ACGCGT,ACGCGG,ACGCGC,ACGCCA,ACGCCT,ACGCCG,
ACGCCC,ACCAAA,ACCAAT,ACCAAG,ACCAAC,ACCATA,ACCATT,ACCATG,ACCATC,A
CCAGA,ACCAGT,ACCAGG,ACCAGC,ACCACA,ACCACT,ACCACG,ACCACC,ACCTAA,A
CCTAT,ACCTAG,ACCTAC,ACCTTA,ACCTTT,ACCTTG,ACCTTC,ACCTGA,ACCTGT,ACC
TGG,ACCTGC,ACCTCA,ACCTCT,ACCTCG,ACCTCC,ACCGAA,ACCGAT,ACCGAG,ACCG
AC,ACCGTA,ACCGTT,ACCGTG,ACCGTC,ACCGGA,ACCGGT,ACCGGG,ACCGGC,ACCGC
A,ACCGCT,ACCGCG,ACCGCC,ACCCAA,ACCCAT,ACCCAG,ACCCAC,ACCCTA,ACCCTT,
ACCCTG,ACCCTC,ACCCGA,ACCCGT,ACCCGG,ACCCGC,ACCCCA,ACCCCT,ACCCCG,A
CCCCC,TAAAAA,TAAAAT,TAAAAG,TAAAAC,TAAATA,TAAATT,TAAATG,TAAATC,TA
AAGA,TAAAGT,TAAAGG,TAAAGC,TAAACA,TAAACT,TAAACG,TAAACC,TAATAA,TA
ATAT,TAATAG,TAATAC,TAATTA,TAATTT,TAATTG,TAATTC,TAATGA,TAATGT,TAAT
GG,TAATGC,TAATCA,TAATCT,TAATCG,TAATCC,TAAGAA,TAAGAT,TAAGAG,TAAGA
C,TAAGTA,TAAGTT,TAAGTG,TAAGTC,TAAGGA,TAAGGT,TAAGGG,TAAGGC,TAAGCA
,TAAGCT,TAAGCG,TAAGCC,TAACAA,TAACAT,TAACAG,TAACAC,TAACTA,TAACTT,T
AACTG,TAACTC,TAACGA,TAACGT,TAACGG,TAACGC,TAACCA,TAACCT,TAACCG,TA
ACCC,TATAAA,TATAAT,TATAAG,TATAAC,TATATA,TATATT,TATATG,TATATC,TATA
GA,TATAGT,TATAGG,TATAGC,TATACA,TATACT,TATACG,TATACC,TATTAA,TATTAT,
TATTAG,TATTAC,TATTTA,TATTTT,TATTTG,TATTTC,TATTGA,TATTGT,TATTGG,TATT
GC,TATTCA,TATTCT,TATTCG,TATTCC,TATGAA,TATGAT,TATGAG,TATGAC,TATGTA,
TATGTT,TATGTG,TATGTC,TATGGA,TATGGT,TATGGG,TATGGC,TATGCA,TATGCT,TA
TGCG,TATGCC,TATCAA,TATCAT,TATCAG,TATCAC,TATCTA,TATCTT,TATCTG,TATCT
C,TATCGA,TATCGT,TATCGG,TATCGC,TATCCA,TATCCT,TATCCG,TATCCC,TAGAAA,T
AGAAT,TAGAAG,TAGAAC,TAGATA,TAGATT,TAGATG,TAGATC,TAGAGA,TAGAGT,TA
GAGG,TAGAGC,TAGACA,TAGACT,TAGACG,TAGACC,TAGTAA,TAGTAT,TAGTAG,TAG
TAC,TAGTTA,TAGTTT,TAGTTG,TAGTTC,TAGTGA,TAGTGT,TAGTGG,TAGTGC,TAGTC
A,TAGTCT,TAGTCG,TAGTCC,TAGGAA,TAGGAT,TAGGAG,TAGGAC,TAGGTA,TAGGTT,
TAGGTG,TAGGTC,TAGGGA,TAGGGT,TAGGGG,TAGGGC,TAGGCA,TAGGCT,TAGGCG,T
AGGCC,TAGCAA,TAGCAT,TAGCAG,TAGCAC,TAGCTA,TAGCTT,TAGCTG,TAGCTC,TA
GCGA,TAGCGT,TAGCGG,TAGCGC,TAGCCA,TAGCCT,TAGCCG,TAGCCC,TACAAA,TAC
AAT,TACAAG,TACAAC,TACATA,TACATT,TACATG,TACATC,TACAGA,TACAGT,TACA
GG,TACAGC,TACACA,TACACT,TACACG,TACACC,TACTAA,TACTAT,TACTAG,TACTAC
,TACTTA,TACTTT,TACTTG,TACTTC,TACTGA,TACTGT,TACTGG,TACTGC,TACTCA,TA

CTCT,TACTCG,TACTCC,TACGAA,TACGAT,TACGAG,TACGAC,TACGTA,TACGTT,TACG TG,TACGTC,TACGGA,TACGGT,TACGGG,TACGGC,TACGCA,TACGCT,TACGCG,TACGC C,TACCAA,TACCAT,TACCAG,TACCAC,TACCTA,TACCTT,TACCTG,TACCTC,TACCGA,T ACCGT,TACCGG,TACCGC,TACCCA,TACCCT,TACCCG,TACCCC,TTAAAA,TTAAAT,TTA AAG,TTAAAC,TTAATA,TTAATT,TTAATG,TTAATC,TTAAGA,TTAAGT,TTAAGG,TTAAG C,TTAACA,TTAACT,TTAACG,TTAACC,TTATAA,TTATAT,TTATAG,TTATAC,TTATTA,T TATTT,TTATTG,TTATTC,TTATGA,TTATGT,TTATGG,TTATGC,TTATCA,TTATCT,TTATC G,TTATCC,TTAGAA,TTAGAT,TTAGAG,TTAGAC,TTAGTA,TTAGTT,TTAGTG,TTAGTC,T TAGGA,TTAGGT,TTAGGG,TTAGGC,TTAGCA,TTAGCT,TTAGCG,TTAGCC,TTACAA,TTA CAT,TTACAG,TTACAC,TTACTA,TTACTT,TTACTG,TTACTC,TTACGA,TTACGT,TTACGG ,TTACGC,TTACCA,TTACCT,TTACCG,TTACCC,TTTAAA,TTTAAT,TTTAAG,TTTAAC,TTT ATA,TTTATT,TTTATG,TTTATC,TTTAGA,TTTAGT,TTTAGG,TTTAGC,TTTACA,TTTACT, TTTACG,TTTACC,TTTTAA,TTTTAT,TTTTAG,TTTTAC,TTTTTA,TTTTTT,TTTTTG,TTTTT C,TTTTGA,TTTTGT,TTTTGG,TTTTGC,TTTTCA,TTTTCT,TTTTCG,TTTTCC,TTTGAA,TTT GAT,TTTGAG,TTTGAC,TTTGTA,TTTGTT,TTTGTG,TTTGTC,TTTGGA,TTTGGT,TTTGGG, TTTGGC,TTTGCA,TTTGCT,TTTGCG,TTTGCC,TTTCAA,TTTCAT,TTTCAG,TTTCAC,TTTC TA,TTTCTT,TTTCTG,TTTCTC,TTTCGA,TTTCGT,TTTCGG,TTTCGC,TTTCCA,TTTCCT,TT TCCG,TTTCCC,TTGAAA,TTGAAT,TTGAAG,TTGAAC,TTGATA,TTGATT,TTGATG,TTGA TC,TTGAGA,TTGAGT,TTGAGG,TTGAGC,TTGACA,TTGACT,TTGACG,TTGACC,TTGTAA, TTGTAT,TTGTAG,TTGTAC,TTGTTA,TTGTTT,TTGTTG,TTGTTC,TTGTGA,TTGTGT,TTGT GG,TTGTGC,TTGTCA,TTGTCT,TTGTCG,TTGTCC,TTGGAA,TTGGAT,TTGGAG,TTGGAC, TTGGTA,TTGGTT,TTGGTG,TTGGTC,TTGGGA,TTGGGT,TTGGGG,TTGGGC,TTGGCA,TT GGCT,TTGGCG,TTGGCC,TTGCAA,TTGCAT,TTGCAG,TTGCAC,TTGCTA,TTGCTT,TTGCT G,TTGCTC,TTGCGA,TTGCGT,TTGCGG,TTGCGC,TTGCCA,TTGCCT,TTGCCG,TTGCCC,T TCAAA,TTCAAT,TTCAAG,TTCAAC,TTCATA,TTCATT,TTCATG,TTCATC,TTCAGA,TTCA GT,TTCAGG,TTCAGC,TTCACA,TTCACT,TTCACG,TTCACC,TTCTAA,TTCTAT,TTCTAG,T TCTAC,TTCTTA,TTCTTT,TTCTTG,TTCTTC,TTCTGA,TTCTGT,TTCTGG,TTCTGC,TTCTC A,TTCTCT,TTCTCG,TTCTCC,TTCGAA,TTCGAT,TTCGAG,TTCGAC,TTCGTA,TTCGTT,TT CGTG,TTCGTC,TTCGGA,TTCGGT,TTCGGG,TTCGGC,TTCGCA,TTCGCT,TTCGCG,TTCGC C,TTCCAA,TTCCAT,TTCCAG,TTCCAC,TTCCTA,TTCCTT,TTCCTG,TTCCTC,TTCCGA,TT CCGT,TTCCGG,TTCCGC,TTCCCA,TTCCCT,TTCCCG,TTCCCC,TGAAAA,TGAAAT,TGAA AG,TGAAAC,TGAATA,TGAATT,TGAATG,TGAATC,TGAAGA,TGAAGT,TGAAGG,TGAA GC,TGAACA,TGAACT,TGAACG,TGAACC,TGATAA,TGATAT,TGATAG,TGATAC,TGATT A,TGATTT,TGATTG,TGATTC,TGATGA,TGATGT,TGATGG,TGATGC,TGATCA,TGATCT,T GATCG,TGATCC,TGAGAA,TGAGAT,TGAGAG,TGAGAC,TGAGTA,TGAGTT,TGAGTG,TG AGTC,TGAGGA,TGAGGT,TGAGGG,TGAGGC,TGAGCA,TGAGCT,TGAGCG,TGAGCC,TG ACAA,TGACAT,TGACAG,TGACAC,TGACTA,TGACTT,TGACTG,TGACTC,TGACGA,TGA CGT,TGACGG,TGACGC,TGACCA,TGACCT,TGACCG,TGACCC,TGTAAA,TGTAAT,TGTA AG,TGTAAC,TGTATA,TGTATT,TGTATG,TGTATC,TGTAGA,TGTAGT,TGTAGG,TGTAGC, TGTACA,TGTACT,TGTACG,TGTACC,TGTTAA,TGTTAT,TGTTAG,TGTTAC,TGTTTA,TGT TTT,TGTTTG,TGTTTC,TGTTGA,TGTTGT,TGTTGG,TGTTGC,TGTTCA,TGTTCT,TGTTCG, TGTTCC,TGTGAA,TGTGAT,TGTGAG,TGTGAC,TGTGTA,TGTGTT,TGTGTG,TGTGTC,TG TGGA,TGTGGT,TGTGGG,TGTGGC,TGTGCA,TGTGCT,TGTGCG,TGTGCC,TGTCAA,TGTC AT,TGTCAG,TGTCAC,TGTCTA,TGTCTT,TGTCTG,TGTCTC,TGTCGA,TGTCGT,TGTCGG, TGTCGC,TGTCCA,TGTCCT,TGTCCG,TGTCCC,TGGAAA,TGGAAT,TGGAAG,TGGAAC,TG GATA,TGGATT,TGGATG,TGGATC,TGGAGA,TGGAGT,TGGAGG,TGGAGC,TGGACA,TGG ACT,TGGACG,TGGACC,TGGTAA,TGGTAT,TGGTAG,TGGTAC,TGGTTA,TGGTTT,TGGTT G,TGGTTC,TGGTGA,TGGTGT,TGGTGG,TGGTGC,TGGTCA,TGGTCT,TGGTCG,TGGTCC,T GGGAA,TGGGAT,TGGGAG,TGGGAC,TGGGTA,TGGGTT,TGGGTG,TGGGTC,TGGGGA,T GGGGT,TGGGGG,TGGGGC,TGGGCA,TGGGCT,TGGGCG,TGGGCC,TGGCAA,TGGCAT,T GGCAG,TGGCAC,TGGCTA,TGGCTT,TGGCTG,TGGCTC,TGGCGA,TGGCGT,TGGCGG,TG GCGC,TGGCCA,TGGCCT,TGGCCG,TGGCCC,TGCAAA,TGCAAT,TGCAAG,TGCAAC,TGC

ATA,TGCATT,TGCATG,TGCATC,TGCAGA,TGCAGT,TGCAGG,TGCAGC,TGCACA,TGCA CT,TGCACG,TGCACC,TGCTAA,TGCTAT,TGCTAG,TGCTAC,TGCTTA,TGCTTT,TGCTTG, TGCTTC,TGCTGA,TGCTGT,TGCTGG,TGCTGC,TGCTCA,TGCTCT,TGCTCG,TGCTCC,TGC GAA,TGCGAT,TGCGAG,TGCGAC,TGCGTA,TGCGTT,TGCGTG,TGCGTC,TGCGGA,TGCG GT,TGCGGG,TGCGGC,TGCGCA,TGCGCT,TGCGCG,TGCGCC,TGCCAA,TGCCAT,TGCCA G,TGCCAC,TGCCTA,TGCCTT,TGCCTG,TGCCTC,TGCCGA,TGCCGT,TGCCGG,TGCCGC,T GCCCA,TGCCCT,TGCCCG,TGCCCC,TCAAAA,TCAAAT,TCAAAG,TCAAAC,TCAATA,TC AATT,TCAATG,TCAATC,TCAAGA,TCAAGT,TCAAGG,TCAAGC,TCAACA,TCAACT,TCA ACG,TCAACC,TCATAA,TCATAT,TCATAG,TCATAC,TCATTA,TCATTT,TCATTG,TCATTC ,TCATGA,TCATGT,TCATGG,TCATGC,TCATCA,TCATCT,TCATCG,TCATCC,TCAGAA,TC AGAT,TCAGAG,TCAGAC,TCAGTA,TCAGTT,TCAGTG,TCAGTC,TCAGGA,TCAGGT,TCA GGG,TCAGGC,TCAGCA,TCAGCT,TCAGCG,TCAGCC,TCACAA,TCACAT,TCACAG,TCAC AC,TCACTA,TCACTT,TCACTG,TCACTC,TCACGA,TCACGT,TCACGG,TCACGC,TCACCA, TCACCT,TCACCG,TCACCC,TCTAAA,TCTAAT,TCTAAG,TCTAAC,TCTATA,TCTATT,TCT ATG,TCTATC,TCTAGA,TCTAGT,TCTAGG,TCTAGC,TCTACA,TCTACT,TCTACG,TCTACC ,TCTTAA,TCTTAT,TCTTAG,TCTTAC,TCTTTA,TCTTTT,TCTTTG,TCTTTC,TCTTGA,TCTT GT,TCTTGG,TCTTGC,TCTTCA,TCTTCT,TCTTCG,TCTTCC,TCTGAA,TCTGAT,TCTGAG,T CTGAC,TCTGTA,TCTGTT,TCTGTG,TCTGTC,TCTGGA,TCTGGT,TCTGGG,TCTGGC,TCTG CA,TCTGCT,TCTGCG,TCTGCC,TCTCAA,TCTCAT,TCTCAG,TCTCAC,TCTCTA,TCTCTT,T CTCTG,TCTCTC,TCTCGA,TCTCGT,TCTCGG,TCTCGC,TCTCCA,TCTCCT,TCTCCG,TCTCC C,TCGAAA,TCGAAT,TCGAAG,TCGAAC,TCGATA,TCGATT,TCGATG,TCGATC,TCGAGA, TCGAGT,TCGAGG,TCGAGC,TCGACA,TCGACT,TCGACG,TCGACC,TCGTAA,TCGTAT,TC GTAG,TCGTAC,TCGTTA,TCGTTT,TCGTTG,TCGTTC,TCGTGA,TCGTGT,TCGTGG,TCGTG C,TCGTCA,TCGTCT,TCGTCG,TCGTCC,TCGGAA,TCGGAT,TCGGAG,TCGGAC,TCGGTA, TCGGTT,TCGGTG,TCGGTC,TCGGGA,TCGGGT,TCGGGG,TCGGGC,TCGGCA,TCGGCT,TC GGCG,TCGGCC,TCGCAA,TCGCAT,TCGCAG,TCGCAC,TCGCTA,TCGCTT,TCGCTG,TCGC TC,TCGCGA,TCGCGT,TCGCGG,TCGCGC,TCGCCA,TCGCCT,TCGCCG,TCGCCC,TCCAAA ,TCCAAT,TCCAAG,TCCAAC,TCCATA,TCCATT,TCCATG,TCCATC,TCCAGA,TCCAGT,TC CAGG,TCCAGC,TCCACA,TCCACT,TCCACG,TCCACC,TCCTAA,TCCTAT,TCCTAG,TCCT AC,TCCTTA,TCCTTT,TCCTTG,TCCTTC,TCCTGA,TCCTGT,TCCTGG,TCCTGC,TCCTCA,T CCTCT,TCCTCG,TCCTCC,TCCGAA,TCCGAT,TCCGAG,TCCGAC,TCCGTA,TCCGTT,TCCG TG,TCCGTC,TCCGGA,TCCGGT,TCCGGG,TCCGGC,TCCGCA,TCCGCT,TCCGCG,TCCGCC, TCCCAA,TCCCAT,TCCCAG,TCCCAC,TCCCTA,TCCCTT,TCCCTG,TCCCTC,TCCCGA,TCC CGT,TCCCGG,TCCCGC,TCCCCA,TCCCCT,TCCCCG,TCCCCC,GAAAAA,GAAAAT,GAAA AG,GAAAAC,GAAATA,GAAATT,GAAATG,GAAATC,GAAAGA,GAAAGT,GAAAGG,GAA AGC,GAAACA,GAAACT,GAAACG,GAAACC,GAATAA,GAATAT,GAATAG,GAATAC,GA ATTA,GAATTT,GAATTG,GAATTC,GAATGA,GAATGT,GAATGG,GAATGC,GAATCA,GAA TCT,GAATCG,GAATCC,GAAGAA,GAAGAT,GAAGAG,GAAGAC,GAAGTA,GAAGTT,GAA GTG,GAAGTC,GAAGGA,GAAGGT,GAAGGG,GAAGGC,GAAGCA,GAAGCT,GAAGCG,GA AGCC,GAACAA,GAACAT,GAACAG,GAACAC,GAACTA,GAACTT,GAACTG,GAACTC,GA ACGA,GAACGT,GAACGG,GAACGC,GAACCA,GAACCT,GAACCG,GAACCC,GATAAA,GA TAAT,GATAAG,GATAAC,GATATA,GATATT,GATATG,GATATC,GATAGA,GATAGT,GAT AGG,GATAGC,GATACA,GATACT,GATACG,GATACC,GATTAA,GATTAT,GATTAG,GATT AC,GATTTA,GATTTT,GATTTG,GATTTC,GATTGA,GATTGT,GATTGG,GATTGC,GATTCA, GATTCT,GATTCG,GATTCC,GATGAA,GATGAT,GATGAG,GATGAC,GATGTA,GATGTT,G ATGTG,GATGTC,GATGGA,GATGGT,GATGGG,GATGGC,GATGCA,GATGCT,GATGCG,G ATGCC,GATCAA,GATCAT,GATCAG,GATCAC,GATCTA,GATCTT,GATCTG,GATCTC,GA TCGA,GATCGT,GATCGG,GATCGC,GATCCA,GATCCT,GATCCG,GATCCC,GAGAAA,GAG AAT,GAGAAG,GAGAAC,GAGATA,GAGATT,GAGATG,GAGATC,GAGAGA,GAGAGT,GA GAGG,GAGAGC,GAGACA,GAGACT,GAGACG,GAGACC,GAGTAA,GAGTAT,GAGTAG,G AGTAC,GAGTTA,GAGTTT,GAGTTG,GAGTTC,GAGTGA,GAGTGT,GAGTGG,GAGTGC,GA GTCA,GAGTCT,GAGTCG,GAGTCC,GAGGAA,GAGGAT,GAGGAG,GAGGAC,GAGGTA,GA

GGTT,GAGGTG,GAGGTC,GAGGGA,GAGGGT,GAGGGG,GAGGGC,GAGGCA,GAGGCT,G
AGGCG,GAGGCC,GAGCAA,GAGCAT,GAGCAG,GAGCAC,GAGCTA,GAGCTT,GAGCTG,G
AGCTC,GAGCGA,GAGCGT,GAGCGG,GAGCGC,GAGCCA,GAGCCT,GAGCCG,GAGCCC,G
ACAAA,GACAAT,GACAAG,GACAAC,GACATA,GACATT,GACATG,GACATC,GACAGA,G
ACAGT,GACAGG,GACAGC,GACACA,GACACT,GACACG,GACACC,GACTAA,GACTAT,G
ACTAG,GACTAC,GACTTA,GACTTT,GACTTG,GACTTC,GACTGA,GACTGT,GACTGG,GA
CTGC,GACTCA,GACTCT,GACTCG,GACTCC,GACGAA,GACGAT,GACGAG,GACGAC,GA
CGTA,GACGTT,GACGTG,GACGTC,GACGGA,GACGGT,GACGGG,GACGGC,GACGCA,GA
CGCT,GACGCG,GACGCC,GACCAA,GACCAT,GACCAG,GACCAC,GACCTA,GACCTT,GA
CCTG,GACCTC,GACCGA,GACCGT,GACCGG,GACCGC,GACCCA,GACCCT,GACCCG,GA
CCCC,GTAAAA,GTAAAT,GTAAAG,GTAAAC,GTAATA,GTAATT,GTAATG,GTAATC,GTA
AGA,GTAAGT,GTAAGG,GTAAGC,GTAACA,GTAACT,GTAACG,GTAACC,GTATAA,GTA
TAT,GTATAG,GTATAC,GTATTA,GTATTT,GTATTG,GTATTC,GTATGA,GTATGT,GTATG
G,GTATGC,GTATCA,GTATCT,GTATCG,GTATCC,GTAGAA,GTAGAT,GTAGAG,GTAGAC,
GTAGTA,GTAGTT,GTAGTG,GTAGTC,GTAGGA,GTAGGT,GTAGGG,GTAGGC,GTAGCA,G
TAGCT,GTAGCG,GTAGCC,GTACAA,GTACAT,GTACAG,GTACAC,GTACTA,GTACTT,GT
ACTG,GTACTC,GTACGA,GTACGT,GTACGG,GTACGC,GTACCA,GTACCT,GTACCG,GTA
CCC,GTTAAA,GTTAAT,GTTAAG,GTTAAC,GTTATA,GTTATT,GTTATG,GTTATC,GTTAG
A,GTTAGT,GTTAGG,GTTAGC,GTTACA,GTTACT,GTTACG,GTTACC,GTTTAA,GTTTAT,G
TTTAG,GTTTAC,GTTTTA,GTTTTT,GTTTTG,GTTTTC,GTTTGA,GTTTGT,GTTTGG,GTTTG
C,GTTTCA,GTTTCT,GTTTCG,GTTTCC,GTTGAA,GTTGAT,GTTGAG,GTTGAC,GTTGTA,G
TTGTT,GTTGTG,GTTGTC,GTTGGA,GTTGGT,GTTGGG,GTTGGC,GTTGCA,GTTGCT,GTT
GCG,GTTGCC,GTTCAA,GTTCAT,GTTCAG,GTTCAC,GTTCTA,GTTCTT,GTTCTG,GTTCTC
,GTTCGA,GTTCGT,GTTCGG,GTTCGC,GTTCCA,GTTCCT,GTTCCG,GTTCCC,GTGAAA,GT
GAAT,GTGAAG,GTGAAC,GTGATA,GTGATT,GTGATG,GTGATC,GTGAGA,GTGAGT,GTG
AGG,GTGAGC,GTGACA,GTGACT,GTGACG,GTGACC,GTGTAA,GTGTAT,GTGTAG,GTGT
AC,GTGTTA,GTGTTT,GTGTTG,GTGTTC,GTGTGA,GTGTGT,GTGTGG,GTGTGC,GTGTCA,
GTGTCT,GTGTCG,GTGTCC,GTGGAA,GTGGAT,GTGGAG,GTGGAC,GTGGTA,GTGGTT,G
TGGTG,GTGGTC,GTGGGA,GTGGGT,GTGGGG,GTGGGC,GTGGCA,GTGGCT,GTGGCG,GT
GGCC,GTGCAA,GTGCAT,GTGCAG,GTGCAC,GTGCTA,GTGCTT,GTGCTG,GTGCTC,GTG
CGA,GTGCGT,GTGCGG,GTGCGC,GTGCCA,GTGCCT,GTGCCG,GTGCCC,GTCAAA,GTCA
AT,GTCAAG,GTCAAC,GTCATA,GTCATT,GTCATG,GTCATC,GTCAGA,GTCAGT,GTCAG
G,GTCAGC,GTCACA,GTCACT,GTCACG,GTCACC,GTCTAA,GTCTAT,GTCTAG,GTCTAC,
GTCTTA,GTCTTT,GTCTTG,GTCTTC,GTCTGA,GTCTGT,GTCTGG,GTCTGC,GTCTCA,GTC
TCT,GTCTCG,GTCTCC,GTCGAA,GTCGAT,GTCGAG,GTCGAC,GTCGTA,GTCGTT,GTCGT
G,GTCGTC,GTCGGA,GTCGGT,GTCGGG,GTCGGC,GTCGCA,GTCGCT,GTCGCG,GTCGCC,
GTCCAA,GTCCAT,GTCCAG,GTCCAC,GTCCTA,GTCCTT,GTCCTG,GTCCTC,GTCCGA,GT
CCGT,GTCCGG,GTCCGC,GTCCCA,GTCCCT,GTCCCG,GTCCCC,GGAAAA,GGAAAT,GGA
AAG,GGAAAC,GGAATA,GGAATT,GGAATG,GGAATC,GGAAGA,GGAAGT,GGAAGG,GG
AAGC,GGAACA,GGAACT,GGAACG,GGAACC,GGATAA,GGATAT,GGATAG,GGATAC,G
GATTA,GGATTT,GGATTG,GGATTC,GGATGA,GGATGT,GGATGG,GGATGC,GGATCA,GG
ATCT,GGATCG,GGATCC,GGAGAA,GGAGAT,GGAGAG,GGAGAC,GGAGTA,GGAGTT,GG
AGTG,GGAGTC,GGAGGA,GGAGGT,GGAGGG,GGAGGC,GGAGCA,GGAGCT,GGAGCG,G
GAGCC,GGACAA,GGACAT,GGACAG,GGACAC,GGACTA,GGACTT,GGACTG,GGACTC,G
GACGA,GGACGT,GGACGG,GGACGC,GGACCA,GGACCT,GGACCG,GGACCC,GGTAAA,G
GTAAT,GGTAAG,GGTAAC,GGTATA,GGTATT,GGTATG,GGTATC,GGTAGA,GGTAGT,GG
TAGG,GGTAGC,GGTACA,GGTACT,GGTACG,GGTACC,GGTTAA,GGTTAT,GGTTAG,GGT
TAC,GGTTTA,GGTTTT,GGTTTG,GGTTTC,GGTTGA,GGTTGT,GGTTGG,GGTTGC,GGTTC
A,GGTTCT,GGTTCG,GGTTCC,GGTGAA,GGTGAT,GGTGAG,GGTGAC,GGTGTA,GGTGTT,
GGTGTG,GGTGTC,GGTGGA,GGTGGT,GGTGGG,GGTGGC,GGTGCA,GGTGCT,GGTGCG,
GGTGCC,GGTCAA,GGTCAT,GGTCAG,GGTCAC,GGTCTA,GGTCTT,GGTCTG,GGTCTC,G
GTCGA,GGTCGT,GGTCGG,GGTCGC,GGTCCA,GGTCCT,GGTCCG,GGTCCC,GGGAAA,GG

GAAT,GGGAAG,GGGAAC,GGGATA,GGGATT,GGGATG,GGGATC,GGGAGA,GGGAGT,G
GGAGG,GGGAGC,GGGACA,GGGACT,GGGACG,GGGACC,GGGTAA,GGGTAT,GGGTAG,
GGGTAC,GGGTTA,GGGTTT,GGGTTG,GGGTTC,GGGTGA,GGGTGT,GGGTGG,GGGTGC,G
GGTCA,GGGTCT,GGGTCG,GGGTCC,GGGGAA,GGGGAT,GGGGAG,GGGGAC,GGGGTA,G
GGGTT,GGGGTG,GGGGTC,GGGGGA,GGGGGT,GGGGGG,GGGGGC,GGGGCA,GGGGCT,
GGGGCG,GGGGCC,GGGCAA,GGGCAT,GGGCAG,GGGCAC,GGGCTA,GGGCTT,GGGCTG,
GGGCTC,GGGCGA,GGGCGT,GGGCGG,GGGCGC,GGGCCA,GGGCCT,GGGCCG,GGGCCC,
GGCAAA,GGCAAT,GGCAAG,GGCAAC,GGCATA,GGCATT,GGCATG,GGCATC,GGCAGA,
GGCAGT,GGCAGG,GGCAGC,GGCACA,GGCACT,GGCACG,GGCACC,GGCTAA,GGCTAT,
GGCTAG,GGCTAC,GGCTTA,GGCTTT,GGCTTG,GGCTTC,GGCTGA,GGCTGT,GGCTGG,G
GCTGC,GGCTCA,GGCTCT,GGCTCG,GGCTCC,GGCGAA,GGCGAT,GGCGAG,GGCGAC,G
GCGTA,GGCGTT,GGCGTG,GGCGTC,GGCGGA,GGCGGT,GGCGGG,GGCGGC,GGCGCA,G
GCGCT,GGCGCG,GGCGCC,GGCCAA,GGCCAT,GGCCAG,GGCCAC,GGCCTA,GGCCTT,G
GCCTG,GGCCTC,GGCCGA,GGCCGT,GGCCGG,GGCCGC,GGCCCA,GGCCCT,GGCCCG,G
GCCCC,GCAAAA,GCAAAT,GCAAAG,GCAAAC,GCAATA,GCAATT,GCAATG,GCAATC,G
CAAGA,GCAAGT,GCAAGG,GCAAGC,GCAACA,GCAACT,GCAACG,GCAACC,GCATAA,G
CATAT,GCATAG,GCATAC,GCATTA,GCATTT,GCATTG,GCATTC,GCATGA,GCATGT,GC
ATGG,GCATGC,GCATCA,GCATCT,GCATCG,GCATCC,GCAGAA,GCAGAT,GCAGAG,GC
AGAC,GCAGTA,GCAGTT,GCAGTG,GCAGTC,GCAGGA,GCAGGT,GCAGGG,GCAGGC,GC
AGCA,GCAGCT,GCAGCG,GCAGCC,GCACAA,GCACAT,GCACAG,GCACAC,GCACTA,GC
ACTT,GCACTG,GCACTC,GCACGA,GCACGT,GCACGG,GCACGC,GCACCA,GCACCT,GCA
CCG,GCACCC,GCTAAA,GCTAAT,GCTAAG,GCTAAC,GCTATA,GCTATT,GCTATG,GCTA
TC,GCTAGA,GCTAGT,GCTAGG,GCTAGC,GCTACA,GCTACT,GCTACG,GCTACC,GCTTA
A,GCTTAT,GCTTAG,GCTTAC,GCTTTA,GCTTTT,GCTTTG,GCTTTC,GCTTGA,GCTTGT,G
CTTGG,GCTTGC,GCTTCA,GCTTCT,GCTTCG,GCTTCC,GCTGAA,GCTGAT,GCTGAG,GCT
GAC,GCTGTA,GCTGTT,GCTGTG,GCTGTC,GCTGGA,GCTGGT,GCTGGG,GCTGGC,GCTG
CA,GCTGCT,GCTGCG,GCTGCC,GCTCAA,GCTCAT,GCTCAG,GCTCAC,GCTCTA,GCTCTT
,GCTCTG,GCTCTC,GCTCGA,GCTCGT,GCTCGG,GCTCGC,GCTCCA,GCTCCT,GCTCCG,G
CTCCC,GCGAAA,GCGAAT,GCGAAG,GCGAAC,GCGATA,GCGATT,GCGATG,GCGATC,G
CGAGA,GCGAGT,GCGAGG,GCGAGC,GCGACA,GCGACT,GCGACG,GCGACC,GCGTAA,G
CGTAT,GCGTAG,GCGTAC,GCGTTA,GCGTTT,GCGTTG,GCGTTC,GCGTGA,GCGTGT,GC
GTGG,GCGTGC,GCGTCA,GCGTCT,GCGTCG,GCGTCC,GCGGAA,GCGGAT,GCGGAG,GC
GGAC,GCGGTA,GCGGTT,GCGGTG,GCGGTC,GCGGGA,GCGGGT,GCGGGG,GCGGGC,GC
GGCA,GCGGCT,GCGGCG,GCGGCC,GCGCAA,GCGCAT,GCGCAG,GCGCAC,GCGCTA,GC
GCTT,GCGCTG,GCGCTC,GCGCGA,GCGCGT,GCGCGG,GCGCGC,GCGCCA,GCGCCT,GCG
CCG,GCGCCC,GCCAAA,GCCAAT,GCCAAG,GCCAAC,GCCATA,GCCATT,GCCATG,GCCA
TC,GCCAGA,GCCAGT,GCCAGG,GCCAGC,GCCACA,GCCACT,GCCACG,GCCACC,GCCTA
A,GCCTAT,GCCTAG,GCCTAC,GCCTTA,GCCTTT,GCCTTG,GCCTTC,GCCTGA,GCCTGT,G
CCTGG,GCCTGC,GCCTCA,GCCTCT,GCCTCG,GCCTCC,GCCGAA,GCCGAT,GCCGAG,GC
CGAC,GCCGTA,GCCGTT,GCCGTG,GCCGTC,GCCGGA,GCCGGT,GCCGGG,GCCGGC,GCC
GCA,GCCGCT,GCCGCG,GCCGCC,GCCCAA,GCCCAT,GCCCAG,GCCCAC,GCCCTA,GCCC
TT,GCCCTG,GCCCTC,GCCCGA,GCCCGT,GCCCGG,GCCCGC,GCCCCA,GCCCCT,GCCCC
G,GCCCCC,CAAAAA,CAAAAT,CAAAAG,CAAAAC,CAAATA,CAAATT,CAAATG,CAAAT
C,CAAAGA,CAAAGT,CAAAGG,CAAAGC,CAAACA,CAAACT,CAAACG,CAAACC,CAATA
A,CAATAT,CAATAG,CAATAC,CAATTA,CAATTT,CAATTG,CAATTC,CAATGA,CAATGT,
CAATGG,CAATGC,CAATCA,CAATCT,CAATCG,CAATCC,CAAGAA,CAAGAT,CAAGAG,
CAAGAC,CAAGTA,CAAGTT,CAAGTG,CAAGTC,CAAGGA,CAAGGT,CAAGGG,CAAGGC,
CAAGCA,CAAGCT,CAAGCG,CAAGCC,CAACAA,CAACAT,CAACAG,CAACAC,CAACTA,
CAACTT,CAACTG,CAACTC,CAACGA,CAACGT,CAACGG,CAACGC,CAACCA,CAACCT,C
AACCG,CAACCC,CATAAA,CATAAT,CATAAG,CATAAC,CATATA,CATATT,CATATG,CA
TATC,CATAGA,CATAGT,CATAGG,CATAGC,CATACA,CATACT,CATACG,CATACC,CAT
TAA,CATTAT,CATTAG,CATTAC,CATTTA,CATTTT,CATTTG,CATTTC,CATTGA,CATTGT

,CATTGG,CATTGC,CATTCA,CATTCT,CATTCG,CATTCC,CATGAA,CATGAT,CATGAG,C
ATGAC,CATGTA,CATGTT,CATGTG,CATGTC,CATGGA,CATGGT,CATGGG,CATGGC,CA
TGCA,CATGCT,CATGCG,CATGCC,CATCAA,CATCAT,CATCAG,CATCAC,CATCTA,CATC
TT,CATCTG,CATCTC,CATCGA,CATCGT,CATCGG,CATCGC,CATCCA,CATCCT,CATCCG,
CATCCC,CAGAAA,CAGAAT,CAGAAG,CAGAAC,CAGATA,CAGATT,CAGATG,CAGATC,
CAGAGA,CAGAGT,CAGAGG,CAGAGC,CAGACA,CAGACT,CAGACG,CAGACC,CAGTAA,
CAGTAT,CAGTAG,CAGTAC,CAGTTA,CAGTTT,CAGTTG,CAGTTC,CAGTGA,CAGTGT,C
AGTGG,CAGTGC,CAGTCA,CAGTCT,CAGTCG,CAGTCC,CAGGAA,CAGGAT,CAGGAG,C
AGGAC,CAGGTA,CAGGTT,CAGGTG,CAGGTC,CAGGGA,CAGGGT,CAGGGG,CAGGGC,C
AGGCA,CAGGCT,CAGGCG,CAGGCC,CAGCAA,CAGCAT,CAGCAG,CAGCAC,CAGCTA,C
AGCTT,CAGCTG,CAGCTC,CAGCGA,CAGCGT,CAGCGG,CAGCGC,CAGCCA,CAGCCT,CA
GCCG,CAGCCC,CACAAA,CACAAT,CACAAG,CACAAC,CACATA,CACATT,CACATG,CAC
ATC,CACAGA,CACAGT,CACAGG,CACAGC,CACACA,CACACT,CACACG,CACACC,CACT
AA,CACTAT,CACTAG,CACTAC,CACTTA,CACTTT,CACTTG,CACTTC,CACTGA,CACTGT,
CACTGG,CACTGC,CACTCA,CACTCT,CACTCG,CACTCC,CACGAA,CACGAT,CACGAG,C
ACGAC,CACGTA,CACGTT,CACGTG,CACGTC,CACGGA,CACGGT,CACGGG,CACGGC,CA
CGCA,CACGCT,CACGCG,CACGCC,CACCAA,CACCAT,CACCAG,CACCAC,CACCTA,CAC
CTT,CACCTG,CACCTC,CACCGA,CACCGT,CACCGG,CACCGC,CACCCA,CACCCT,CACC
CG,CACCCC,CTAAAA,CTAAAT,CTAAAG,CTAAAC,CTAATA,CTAATT,CTAATG,CTAAT
C,CTAAGA,CTAAGT,CTAAGG,CTAAGC,CTAACA,CTAACT,CTAACG,CTAACC,CTATAA,
CTATAT,CTATAG,CTATAC,CTATTA,CTATTT,CTATTG,CTATTC,CTATGA,CTATGT,CTA
TGG,CTATGC,CTATCA,CTATCT,CTATCG,CTATCC,CTAGAA,CTAGAT,CTAGAG,CTAGA
C,CTAGTA,CTAGTT,CTAGTG,CTAGTC,CTAGGA,CTAGGT,CTAGGG,CTAGGC,CTAGCA,
CTAGCT,CTAGCG,CTAGCC,CTACAA,CTACAT,CTACAG,CTACAC,CTACTA,CTACTT,CT
ACTG,CTACTC,CTACGA,CTACGT,CTACGG,CTACGC,CTACCA,CTACCT,CTACCG,CTAC
CC,CTTAAA,CTTAAT,CTTAAG,CTTAAC,CTTATA,CTTATT,CTTATG,CTTATC,CTTAGA,
CTTAGT,CTTAGG,CTTAGC,CTTACA,CTTACT,CTTACG,CTTACC,CTTTAA,CTTTAT,CTT
TAG,CTTTAC,CTTTTA,CTTTTT,CTTTTG,CTTTTC,CTTTGA,CTTTGT,CTTTGG,CTTTGC,C
TTTCA,CTTTCT,CTTTCG,CTTTCC,CTTGAA,CTTGAT,CTTGAG,CTTGAC,CTTGTA,CTTG
TT,CTTGTG,CTTGTC,CTTGGA,CTTGGT,CTTGGG,CTTGGC,CTTGCA,CTTGCT,CTTGCG,
CTTGCC,CTTCAA,CTTCAT,CTTCAG,CTTCAC,CTTCTA,CTTCTT,CTTCTG,CTTCTC,CTTC
GA,CTTCGT,CTTCGG,CTTCGC,CTTCCA,CTTCCT,CTTCCG,CTTCCC,CTGAAA,CTGAAT,
CTGAAG,CTGAAC,CTGATA,CTGATT,CTGATG,CTGATC,CTGAGA,CTGAGT,CTGAGG,C
TGAGC,CTGACA,CTGACT,CTGACG,CTGACC,CTGTAA,CTGTAT,CTGTAG,CTGTAC,CTG
TTA,CTGTTT,CTGTTG,CTGTTC,CTGTGA,CTGTGT,CTGTGG,CTGTGC,CTGTCA,CTGTCT,
CTGTCG,CTGTCC,CTGGAA,CTGGAT,CTGGAG,CTGGAC,CTGGTA,CTGGTT,CTGGTG,CT
GGTC,CTGGGA,CTGGGT,CTGGGG,CTGGGC,CTGGCA,CTGGCT,CTGGCG,CTGGCC,CTG
CAA,CTGCAT,CTGCAG,CTGCAC,CTGCTA,CTGCTT,CTGCTG,CTGCTC,CTGCGA,CTGCG
T,CTGCGG,CTGCGC,CTGCCA,CTGCCT,CTGCCG,CTGCCC,CTCAAA,CTCAAT,CTCAAG,
CTCAAC,CTCATA,CTCATT,CTCATG,CTCATC,CTCAGA,CTCAGT,CTCAGG,CTCAGC,CT
CACA,CTCACT,CTCACG,CTCACC,CTCTAA,CTCTAT,CTCTAG,CTCTAC,CTCTTA,CTCTT
T,CTCTTG,CTCTTC,CTCTGA,CTCTGT,CTCTGG,CTCTGC,CTCTCA,CTCTCT,CTCTCG,CT
CTCC,CTCGAA,CTCGAT,CTCGAG,CTCGAC,CTCGTA,CTCGTT,CTCGTG,CTCGTC,CTCG
GA,CTCGGT,CTCGGG,CTCGGC,CTCGCA,CTCGCT,CTCGCG,CTCGCC,CTCCAA,CTCCAT
,CTCCAG,CTCCAC,CTCCTA,CTCCTT,CTCCTG,CTCCTC,CTCCGA,CTCCGT,CTCCGG,CT
CCGC,CTCCCA,CTCCCT,CTCCCG,CTCCCC,CGAAAA,CGAAAT,CGAAAG,CGAAAC,CGA
ATA,CGAATT,CGAATG,CGAATC,CGAAGA,CGAAGT,CGAAGG,CGAAGC,CGAACA,CGA
ACT,CGAACG,CGAACC,CGATAA,CGATAT,CGATAG,CGATAC,CGATTA,CGATTT,CGAT
TG,CGATTC,CGATGA,CGATGT,CGATGG,CGATGC,CGATCA,CGATCT,CGATCG,CGATC
C,CGAGAA,CGAGAT,CGAGAG,CGAGAC,CGAGTA,CGAGTT,CGAGTG,CGAGTC,CGAGG
A,CGAGGT,CGAGGG,CGAGGC,CGAGCA,CGAGCT,CGAGCG,CGAGCC,CGACAA,CGACA
T,CGACAG,CGACAC,CGACTA,CGACTT,CGACTG,CGACTC,CGACGA,CGACGT,CGACGG

,CGACGC,CGACCA,CGACCT,CGACCG,CGACCC,CGTAAA,CGTAAT,CGTAAG,CGTAAC,
CGTATA,CGTATT,CGTATG,CGTATC,CGTAGA,CGTAGT,CGTAGG,CGTAGC,CGTACA,C
GTACT,CGTACG,CGTACC,CGTTAA,CGTTAT,CGTTAG,CGTTAC,CGTTTA,CGTTTT,CGT
TTG,CGTTTC,CGTTGA,CGTTGT,CGTTGG,CGTTGC,CGTTCA,CGTTCT,CGTTCG,CGTTCC
,CGTGAA,CGTGAT,CGTGAG,CGTGAC,CGTGTA,CGTGTT,CGTGTG,CGTGTC,CGTGGA,C
GTGGT,CGTGGG,CGTGGC,CGTGCA,CGTGCT,CGTGCG,CGTGCC,CGTCAA,CGTCAT,CG
TCAG,CGTCAC,CGTCTA,CGTCTT,CGTCTG,CGTCTC,CGTCGA,CGTCGT,CGTCGG,CGTC
GC,CGTCCA,CGTCCT,CGTCCG,CGTCCC,CGGAAA,CGGAAT,CGGAAG,CGGAAC,CGGAT
A,CGGATT,CGGATG,CGGATC,CGGAGA,CGGAGT,CGGAGG,CGGAGC,CGGACA,CGGAC
T,CGGACG,CGGACC,CGGTAA,CGGTAT,CGGTAG,CGGTAC,CGGTTA,CGGTTT,CGGTTG,
CGGTTC,CGGTGA,CGGTGT,CGGTGG,CGGTGC,CGGTCA,CGGTCT,CGGTCG,CGGTCC,C
GGGAA,CGGGAT,CGGGAG,CGGGAC,CGGGTA,CGGGTT,CGGGTG,CGGGTC,CGGGGA,C
GGGGT,CGGGGG,CGGGGC,CGGGCA,CGGGCT,CGGGCG,CGGGCC,CGGCAA,CGGCAT,C
GGCAG,CGGCAC,CGGCTA,CGGCTT,CGGCTG,CGGCTC,CGGCGA,CGGCGT,CGGCGG,C
GGCGC,CGGCCA,CGGCCT,CGGCCG,CGGCCC,CGCAAA,CGCAAT,CGCAAG,CGCAAC,C
GCATA,CGCATT,CGCATG,CGCATC,CGCAGA,CGCAGT,CGCAGG,CGCAGC,CGCACA,CG
CACT,CGCACG,CGCACC,CGCTAA,CGCTAT,CGCTAG,CGCTAC,CGCTTA,CGCTTT,CGCT
TG,CGCTTC,CGCTGA,CGCTGT,CGCTGG,CGCTGC,CGCTCA,CGCTCT,CGCTCG,CGCTCC,
CGCGAA,CGCGAT,CGCGAG,CGCGAC,CGCGTA,CGCGTT,CGCGTG,CGCGTC,CGCGGA,C
GCGGT,CGCGGG,CGCGGC,CGCGCA,CGCGCT,CGCGCG,CGCGCC,CGCCAA,CGCCAT,C
GCCAG,CGCCAC,CGCCTA,CGCCTT,CGCCTG,CGCCTC,CGCCGA,CGCCGT,CGCCGG,CG
CCGC,CGCCCA,CGCCCT,CGCCCG,CGCCCC,CCAAAA,CCAAAT,CCAAAG,CCAAAC,CCA
ATA,CCAATT,CCAATG,CCAATC,CCAAGA,CCAAGT,CCAAGG,CCAAGC,CCAACA,CCAA
CT,CCAACG,CCAACC,CCATAA,CCATAT,CCATAG,CCATAC,CCATTA,CCATTT,CCATTG
,CCATTC,CCATGA,CCATGT,CCATGG,CCATGC,CCATCA,CCATCT,CCATCG,CCATCC,CC
AGAA,CCAGAT,CCAGAG,CCAGAC,CCAGTA,CCAGTT,CCAGTG,CCAGTC,CCAGGA,CC
AGGT,CCAGGG,CCAGGC,CCAGCA,CCAGCT,CCAGCG,CCAGCC,CCACAA,CCACAT,CC
ACAG,CCACAC,CCACTA,CCACTT,CCACTG,CCACTC,CCACGA,CCACGT,CCACGG,CCA
CGC,CCACCA,CCACCT,CCACCG,CCACCC,CCTAAA,CCTAAT,CCTAAG,CCTAAC,CCTAT
A,CCTATT,CCTATG,CCTATC,CCTAGA,CCTAGT,CCTAGG,CCTAGC,CCTACA,CCTACT,C
CTACG,CCTACC,CCTTAA,CCTTAT,CCTTAG,CCTTAC,CCTTTA,CCTTTT,CCTTTG,CCTT
TC,CCTTGA,CCTTGT,CCTTGG,CCTTGC,CCTTCA,CCTTCT,CCTTCG,CCTTCC,CCTGAA,C
CTGAT,CCTGAG,CCTGAC,CCTGTA,CCTGTT,CCTGTG,CCTGTC,CCTGGA,CCTGGT,CCT
GGG,CCTGGC,CCTGCA,CCTGCT,CCTGCG,CCTGCC,CCTCAA,CCTCAT,CCTCAG,CCTCA
C,CCTCTA,CCTCTT,CCTCTG,CCTCTC,CCTCGA,CCTCGT,CCTCGG,CCTCGC,CCTCCA,C
CTCCT,CCTCCG,CCTCCC,CCGAAA,CCGAAT,CCGAAG,CCGAAC,CCGATA,CCGATT,CC
GATG,CCGATC,CCGAGA,CCGAGT,CCGAGG,CCGAGC,CCGACA,CCGACT,CCGACG,CC
GACC,CCGTAA,CCGTAT,CCGTAG,CCGTAC,CCGTTA,CCGTTT,CCGTTG,CCGTTC,CCGT
GA,CCGTGT,CCGTGG,CCGTGC,CCGTCA,CCGTCT,CCGTCG,CCGTCC,CCGGAA,CCGGA
T,CCGGAG,CCGGAC,CCGGTA,CCGGTT,CCGGTG,CCGGTC,CCGGGA,CCGGGT,CCGGGG
,CCGGGC,CCGGCA,CCGGCT,CCGGCG,CCGGCC,CCGCAA,CCGCAT,CCGCAG,CCGCAC,
CCGCTA,CCGCTT,CCGCTG,CCGCTC,CCGCGA,CCGCGT,CCGCGG,CCGCGC,CCGCCA,C
CGCCT,CCGCCG,CCGCCC,CCCAAA,CCCAAT,CCCAAG,CCCAAC,CCCATA,CCCATT,CC
CATG,CCCATC,CCCAGA,CCCAGT,CCCAGG,CCCAGC,CCCACA,CCCACT,CCCACG,CCC
ACC,CCCTAA,CCCTAT,CCCTAG,CCCTAC,CCCTTA,CCCTTT,CCCTTG,CCCTTC,CCCTGA
,CCCTGT,CCCTGG,CCCTGC,CCCTCA,CCCTCT,CCCTCG,CCCTCC,CCCGAA,CCCGAT,CC
CGAG,CCCGAC,CCCGTA,CCCGTT,CCCGTG,CCCGTC,CCCGGA,CCCGGT,CCCGGG,CCC
GGC,CCCGCA,CCCGCT,CCCGCG,CCCGCC,CCCCAA,CCCCAT,CCCCAG,CCCCAC,CCCC
TA,CCCCTT,CCCCTG,CCCCTC,CCCCGA,CCCCGT,CCCCGG,CCCCGC,CCCCCA,CCCCCT,
CCCCCG,CCCCCC,sd,dfa,hurst,sampen,ac,rvntsl,ac_200,ac_300,label
